# The Anti-Proliferative Activity of the Hybrid TMS-TMF-4f Compound Against Human Cervical Cancer Involves Apoptosis Mediated by STAT3 Inactivation

**DOI:** 10.3390/cancers11121927

**Published:** 2019-12-03

**Authors:** Joo Young Hong, Kyung-Sook Chung, Ji-Sun Shin, Jeong-Hun Lee, Hyo-Sun Gil, Hwi-Ho Lee, Eunwoo Choi, Jung-Hye Choi, Ahmed H.E. Hassan, Yong Sup Lee, Kyung-Tae Lee

**Affiliations:** 1 Department of Pharmaceutical Biochemistry, College of Pharmacy, Kyung Hee University, 26, Kyungheedae-ro, Seoul 02447, Korea; hjisuk1206@naver.com (J.Y.H.); adella76@hanmail.net (K.-S.C.); jsshin@khu.ac.kr (J.-S.S.); ztztzt08@hanmail.net (J.-H.L.); hhlee4083@naver.com (H.-H.L.); 2 Department of Life and Nanopharmaceutical Sciences, Graduate School, Kyung Hee University, 26, Kyungheedae-ro, Seoul 02447, Korea; inaraidmschoi@hanmail.net (E.C.); jchoi@khu.ac.kr (J.-H.C.); kyslee@khu.ac.kr (Y.S.L.); 3 Oriental Pharmaceutical Science, College of Pharmacy, Kyung Hee University, 26, Kyungheedae-ro, Seoul 02447, Korea; 4 Medicinal Chemistry Laboratory, College of Pharmacy, Kyung Hee University, 26, Kyungheedae-ro, Seoul 02447, Korea; ahmed_hassan@mans.edu.eg; 5 Department of Medicinal Chemistry, Faculty of Pharmacy, Mansoura University, Mansoura 35516, Egypt; 6 KHU-KIST Department of Converging Science and Technology, Kyung Hee University, 26, Kyungheedae-ro, Seoul 02447, Korea

**Keywords:** TMS-TMF-4f, human cervical cancer, STAT3, apoptosis, xenograft

## Abstract

We previously reported the potential anti-proliferative activity of 3-(5,6,7-trimethoxy-4-oxo-4*H*-chromen-2-yl)-*N*-(3,4,5-trimethoxyphenyl) benzamide (TMS-TMF-4f) against human cancer cells; however, the underlying molecular mechanisms have not been investigated. In the present study, TMS-TMF-4f showed the highest cytotoxicity in human cervical cancer cells (HeLa and CaSki) and low cytotoxicity in normal ovarian epithelial cells. Annexin V-FITC and propidium iodide (PI) double staining revealed that TMS-TMF-4f-induced cytotoxicity was caused by the induction of apoptosis in both HeLa and CaSki cervical cancer cells. The compound TMS-TMF-4f enhanced the activation of caspase-3, caspase-8, and caspase-9 and regulated Bcl-2 family proteins, which led to mitochondrial membrane potential (MMP) loss and resulted in the release of cytochrome *c* and Smac/DIABLO into the cytosol. Also, TMS-TMF-4f suppressed both constitutive and IL-6-inducible levels of phosphorylated STAT3 (p-STAT3) and associated proteins such as Mcl-1, cyclin D1, survivin, and c-Myc in both cervical cancer cells. STAT-3 overexpression completely ameliorated TMS-TMF-4f-induced apoptotic cell death and PARP cleavage. Docking analysis revealed that TMS-TMF-4f could bind to unphosphorylated STAT3 and inhibit its interconversion to the activated form. Notably, intraperitoneal administration of TMS-TMF-4f (5, 10, or 20 mg/kg) decreased tumor growth in a xenograft cervical cancer mouse model, demonstrated by the increase in TUNEL staining and PARP cleavage and the reduction in p-STAT3, Mcl-1, cyclin D1, survivin, and c-Myc expression levels in tumor tissues. Taken together, our results suggest that TMS-TMF-4f may potentially inhibit human cervical tumor growth through the induction of apoptosis via STAT3 suppression.

## 1. Introduction

Cervical cancer is a major gynecologic cancer worldwide, ranked as the fourth most frequently occurring cancer among women and characterized by a 30% mortality rate within five years of treatment [1]. Human papillomavirus (HPV) infection is observed in more than 75% of cases [2]; however, most individuals who have had HPV infection do not develop cervical cancer. Cervical cancer typically develops from precancerous changes over 10 to 20 years [3]. Around 90% of cervical cancer cases are squamous cell carcinomas, and 10% of cases are adenocarcinomas with a small number of cases involving other types of carcinomas [4]. Although standard treatments such as surgery and combination of chemotherapy and radiotherapy have significantly improved the clinical outcomes of cervical cancer patients in early stages, the clinical management of cervical cancer in advanced stages remains a challenge. To date, chemotherapies using platinum and taxol are still standard therapeutic approaches; however, the outcomes are not satisfactory, especially for patients with chemoresistance [1]. Hence, the demand for new drugs with high efficacy and low adverse effects has increased. The use of natural products might allow the development of effective and safer anti-cancer drugs [5].

Programmed cell death (PCD) is a normal process which is a vital component of many natural processes of the body, such as cell turnover, appropriate formation, and function of the immune system while any disorder in this process can be an important factor in several types of cancer [6]. Nevertheless, apoptosis remains the most well-known and studied for PCD. There are two different molecular mechanisms controlling apoptosis, the intrinsic pathway initiated by intracellular stimuli such as DNA damage, and the extrinsic pathway initiated by extracellular death receptors; both pathways depend on the activation of a series of cysteine-aspartic proteases known as caspases [7]. Caspase protease activity is essential for apoptosis; once active, caspases cleave hundreds of different proteins leading to rapid cell death with distinctive biochemical and morphological hallmarks, including DNA fragmentation, destruction of the nuclear proteins and cytoskeleton, crosslinking of proteins, the expression of ligands for phagocytic cells and the formation of apoptotic bodies [8]. Therefore, focusing on the caspase-dependent apoptosis pathway can be an appropriate target for the treatment of many cancers.

Signal transducer and activator of transcription (STAT) proteins are a family of transcription factors including STAT1, STAT2, STAT3, STAT4, STAT5a, STAT5b, and STAT6 [9]. STAT3 regulates critical functions in normal and malignant human tissues, such as differentiation, proliferation, survival, angiogenesis, and immune system regulation [10]. Under normal conditions, STAT3 activation is tightly regulated by the presence or absence of polypeptide ligands bound to its receptor. However, in cancer cells, cytokine and growth factor receptors become constitutively activated, most commonly by the autocrine or paracrine expression of their respective ligands such as interleukin 6 (IL-6) and epidermal growth factor (EGF) [11]. The STAT3 signaling cascade is triggered by upstream kinase signals such as Janus kinase (JAK) and Src, resulting in the phosphorylation and activation of STAT3 monomers. Phosphorylated STAT3 (p-STAT3) monomers form dimers and translocate into the nucleus, bind to specific DNA response elements, and induce the transcription of STAT3-targeted gene products involved in tumor cell proliferation, angiogenesis, metastasis, and immunoediting [9]. Consequently, approximately 70% of human solid and hematological tumors display overexpressed or constitutively active STAT3. It has been reported that disruption of constitutively activated STAT3 can promote cell apoptosis, and the STAT3-signaling pathway has become an attractive key target for cancer therapy [12].

The molecular hybridization of anti-cancer agents is an effective strategy to generate novel ligands with improved anti-cancer activity relative to the parent molecules [13,14,15,16]. By adopting this strategy, we recently reported the synthesis of natural product hybrids that could induce potential anti-proliferative activity [16]. We hybridized 3,5,4′-trimethoxystilbene (TMS), which is a permethylated analog of resveratrol, with 5,6,7-trimethoxyflavone (TMF), which is a flavone derivative whose anti-cancer activity is triggered by nuclear factor-kappa B (NF-кB) suppression [17,18,19]. It is well known that resveratrol has been the subject of several biological evaluations, as well as clinical trials as a potential anticancer agent impacting several pathways that control the proliferation and survival of cells [19]. Nevertheless, the clinical benefit of resveratrol is not sufficiently confirmed and it has poor pharmacokinetics, as well as chemical instabilities that hinder further development [20,21]. To overcome these problems, the molecular hybridization of anti-cancer candidates is suggested as a new solution. In our previous study, the synthesized chimeric molecules were screened for their anti-proliferative activity against nine cancer cells [19]. However, the molecular mechanisms involved remain unclear. In this study, we evaluated the effect of these synthetic hybrids on the viability of other five human cancer cells and investigated their underlying molecular mechanisms, which may be mediated by the suppression of STAT3 activation in human cervical cancer cells in vitro and in vivo.

## 2. Results

### 2.1. TMS-TMF-4f Suppresses the Proliferation of HeLa and CaSki Human Cervical Cancer Cells by Inducing Caspase-Dependent Apoptosis

To determine the effects of six hybrid TMS-TMF compounds (**4a**–**4f**) on growth inhibition, we measured cell viability by MTT assay for four human cancer cell lines (HCT116, human colorectal carcinoma; A549, human lung carcinoma; AsPC-1, human pancreatic adenocarcinoma; and HeLa, human cervical carcinoma). The chemical structures and calculated half-maximal inhibitory concentration (IC_50_) values of six hybrid analogs are listed in Table 1.

**Figure 1:**
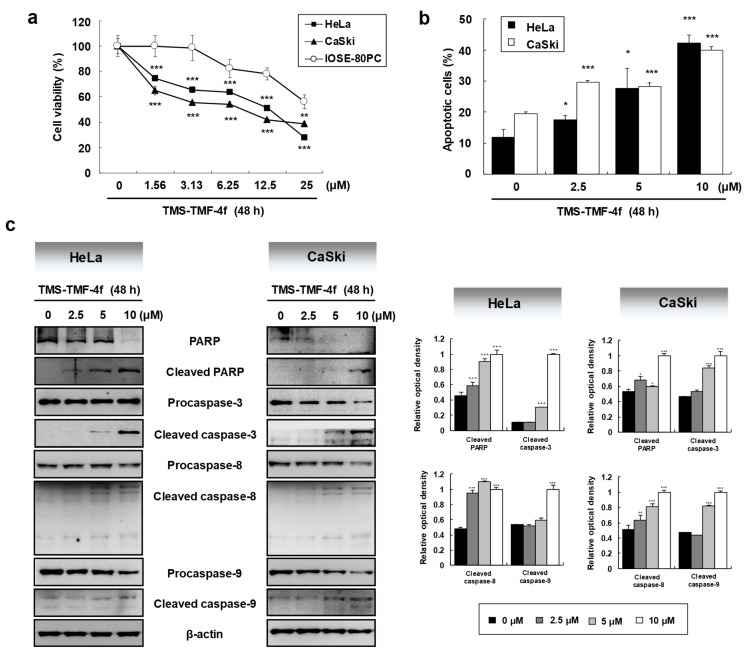

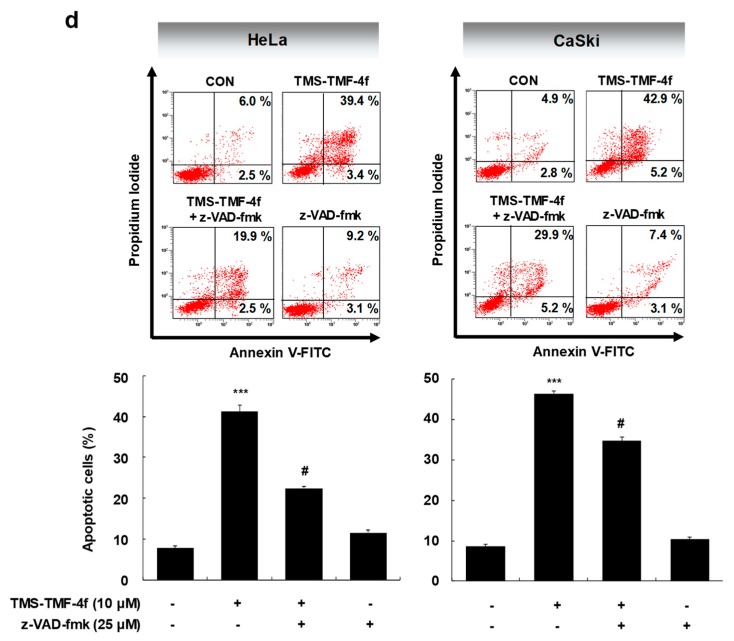
**3**-(5,6,7-trimethoxy-4-oxo-4*H*-chromen-2-yl)-*N*-(3,4,5-trimethoxyphenyl) benzamide (TMS-TMF-4f)-induced cytotoxicity and apoptosis mediated by caspase activation in human cervical cancer cells. (**a**) Cells were treated with various concentrations of TMS-TMF-4f for 48 h. The cell viability of each cell was determined by MTT assay. (**b**) HeLa and CaSki cells were treated with the indicated concentrations (0, 2.5, 5, or 10 μM) of TMS-TMF-4f for 48 h and co-stained with annexin V-FITC and propidium iodide (PI) for detecting apoptosis by flow cytometry. (**c**) HeLa and CaSki cells were treated with TMS-TMF-4f (2.5, 5, or 10 μM), and Western blotting was performed to examine the expression level of proteins. The relative optical density ratio was determined using a densitometric analysis program (Bio-Rad Quantity One^®^ Software, ver 4.6.3 (Basic), Bio-Rad Laboratories Inc., Hercules, CA, USA), normalized to β-actin. (**d**) Cells were pretreated with the broad caspase inhibitor z-VAD-fmk (25 μM) for 30 min, followed by treatment with 10 μM TMS-TMF-4f for 48 h. The cells were co-stained with annexin V-FITC and PI, and apoptosis was detected by flow cytometry. Data are presented as the mean ± SD of three independent experiments. * *p* < 0.05, ** *p* < 0.01, *** *p* < 0.001 vs. the control group. # *p* < 0.05 vs. the TMS-TMF-4f-treated group.

Among these compounds, TMS-TMF-4f showed strong cytotoxic effects on four human cancer cell lines with the lowest IC_50_ value against HeLa human cervical cancer cells. Therefore, we further investigated the cytotoxic effects of TMS-TMF-4f on human cervical cancer and normal ovarian epithelial cells. As shown in Figure 1a, MTT assay revealed that the cytotoxic effect of TMS-TMF-4f was more potent on HeLa and CaSki cervical cancer cells (IC_50_: 12.07 ± 1.84 μM and 9.52 ± 1.27 μM, respectively) than on IOSE-80PC normal ovarian epithelial cells (IC_50_: 33.27 ± 2.29 μM). In addition, to investigate whether the cytotoxicity of TMS-TMF-4f is associated with apoptosis, we performed PI and annexin V-FITC double staining. After treatment with TMS-TMF-4f for 48 h, annexin V-positive cells were significantly increased in a concentration-dependent manner, suggesting that TMS-TMF-4f may induce cervical cancer cell death via apoptosis rather than non-specific necrosis (Figure 1b).

As the activation of caspases occurs via caspase cleavage and initiates the apoptosis cascade, we investigated the involvement of caspase activation in TMS-TMF-4f-induced apoptosis in HeLa and CaSki cells by western blot analysis. As shown in Figure 1c, TMS-TMF-4f increased cleaved PARP, caspase-3, caspase-8, and caspase-9 in a concentration-dependent manner in human cervical cancer cells. To further determine the contribution of caspase to TMS-TMF-4f-induced apoptosis, HeLa and CaSki cells were pretreated with 25 μM z-VAD-fmk, a broad caspase inhibitor, to counteract caspase activation. As shown in Figure 1d, z-VAD-fmk pretreatment significantly reduced TMS-TMF-4f-induced apoptotic HeLa and CaSki cells by 22.46 ± 0.51% (vs. 42.82 ± 1.65%, *p* < 0.001) and 35.17 ± 1.06% (vs. 48.15 ± 0.59%, *p* < 0.001), respectively, suggesting that TMS-TMF-4f-induced apoptosis may be partly dependent on caspase activation.

### 2.2. TMS-TMF-4f Induces Mitochondria-Dependent Apoptosis by Regulating Bcl-2 Family Proteins

**Figure 2:**
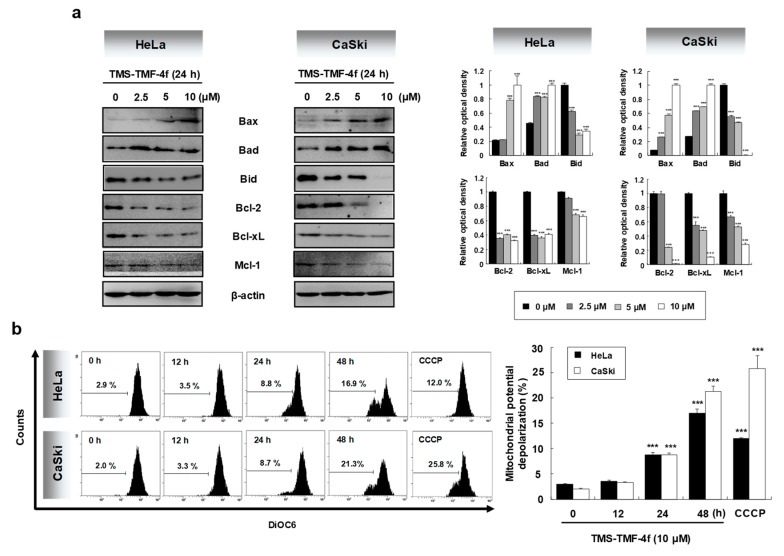

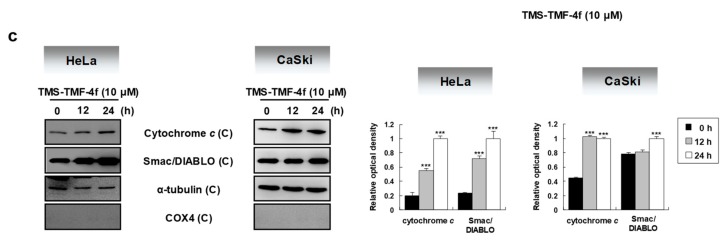
TMS-TMF-4f disrupts the mitochondrial membrane potential (MMP) in human cervical cancer cells. (**a**) HeLa and CaSki cells were treated with the indicated concentrations (0, 2.5, 5, or 10 μM) of TMS-TMF-4f for 24 h. Total cellular proteins were prepared, resolved by SDS-PAGE, and detected using specific Bax, Bad, Bid, Bcl-2, Bcl-xL, and Mcl-1 antibodies. β-actin was used as an internal control. (**b**) After treatment with 10 μM TMS-TMF-4f for the indicated times, cells were stained with DiOC6 (30 μM) for 30 min and detected by flow cytometry. CCCP (100 µM) was used as a positive control. The marker shows the area of the cell population used for analysis. (**c**) Cells were harvested, and cytosolic fractions were isolated using a mitochondrial fractionation kit. Cytosolic proteins were prepared, resolved by SDS-PAGE, and detected using specific cytochrome *c* and Smac/DIABLO antibodies. α-tubulin was used as an internal control. The relative optical density ratio was determined using a densitometric analysis program (Bio-Rad Quantity One^®^ Software, version 4.6.3 (Basic)), normalized to the internal control. Data are presented as the mean ± SD of three independent experiments. *** *p* < 0.001 vs. the control group.

Caspase-dependent apoptosis is regulated through two major signaling pathways, the intrinsic and extrinsic pathways [22]. In both apoptotic pathways, mitochondria plays a central role, dependent on the levels of Bcl-2 family proteins [23]. In this study, the expression levels of Bcl-2 family proteins in TMS-TMF-4f-treated cervical cancer cells were determined by Western blot analysis. TMS-TMF-4f markedly increased the expression levels of pro-apoptotic Bcl-2 family proteins such as Bax and Bad and induced the cleavage of Bid, and it downregulated the expression levels of anti-apoptotic Bcl-2 family proteins such as Bcl-2, Bcl-xL, and Mcl-1 (Figure 2a). Members of the pro-apoptotic Bcl-2 family translocate to the outer mitochondrial membrane where they oligomerize and alter the mitochondrial membrane potential (MMP), leading to the opening of the mitochondrial permeability transition pore (mPTP) [24]. Cytochrome *c* and Smac/DIABLO are released from the mitochondria into the cytoplasm through the opened mPTP and form a complex with caspase-9 and Apaf-1 (known as the apoptosome) and inhibitor of apoptosis proteins (IAPs), respectively [25]. Therefore, we examined changes in the MMP and the release of cytochrome *c* and Smac/DIABLO into the cytosol of TMS-TMF-4f-treated cervical cancer cells. To evaluate MMP changes in HeLa and CaSki cells following exposure to TMS-TMF-4f, we used DiOC6, a mitochondria-specific and voltage-dependent dye. Following the treatment of these cells with TMS-TMF-4f (10 μM), mitochondrial membrane depolarization was significantly induced by 16.9% and 21.3% in HeLa and CaSki cells at 48 h, respectively (Figure 2b). It is known that a decrease in the MMP is preceded or accompanied by the release of cytochrome *c* and Smac/DIABLO into the cytosol; thus, we measured cytochrome *c* and Smac/DIABLO levels in cytosolic proteins by Western blot analysis. Indeed, the release of cytochrome *c* and Smac/DIABLO from the mitochondria to cytosol was observed in TMS-TMF-4f-treated human cervical cancer cells (Figure 2c). Collectively, our results showed that TMS-TMF could induce MMP loss and the translocation of mitochondrial cytochrome *c* and Smac/DIABLO to the cytosol via unbalanced Bcl-2 family protein expression, which may mediate TMS-TMF-stimulated apoptosis.

### 2.3. STAT3 is Involved in TMS-TMF-4f-Induced Apoptosis

**Figure 3:**
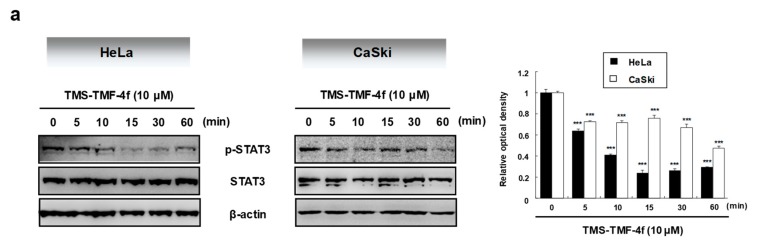

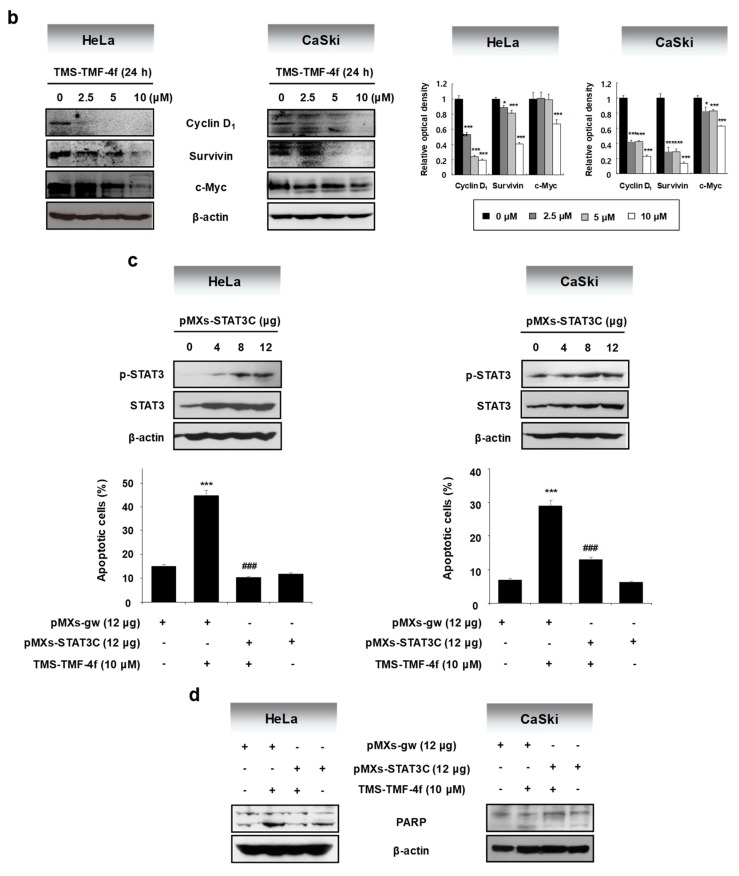
TMS-TMF-4f suppresses the STAT3 activation and STAT3-related protein expression in human cervical cancer cells. (**a**) After treatment with 10 μM TMS-TMF-4f for the indicated times (5, 10, 15, 30, and 60 min), total cellular proteins were prepared, resolved by SDS-PAGE, and detected using specific p-STAT3 and STAT3 antibodies. β-actin was used an internal control. (**b**) Cells were treated with 10 μM TMS-TMF-4f for 24 h. Total cellular proteins were prepared, resolved by SDS-PAGE, and detected using specific cyclin D_1_, survivin, and c-Myc antibodies. β-actin was used an internal control. The relative optical density ratio was determined using a densitometric analysis program (Bio-Rad Quantity One^®^ Software, version 4.6.3 (Basic)), normalized to the internal control. After transfection with pMXs-STAT3C (12 μg), the cells were treated with 10 μM TMS-TMF-4f for 24 h. (**c**) Annexin V-FITC and PI staining and (**d**) Western blot analyses were performed to detect apoptotic cell death. Data are presented as the mean ± SD of three independent experiments. *** *p* < 0.001 vs. the pMXs-gw-transfected control group. ### *p* < 0.001 vs. the TMS-TMF-4f-treated control group.

As the aberrant activation of the key transcription factor STAT3 is associated with cell survival and proliferation [26], we examined the effects of TMS-TMF-4f on STAT3 activation in cervical cancer cells. Our results showed that TMS-TMF-4f decreased p-STAT3 levels and STAT3-mediated anti-apoptotic proteins including cyclin D_1_, survivin, and c-Myc in both HeLa and CaSki cervical cancer cells (Figure 3a,b). However, the levels of unphosphorylated STAT3 were unaffected by TMS-TMF-4f. To further elucidate the role of STAT3 in TMS-TMF-4f-induced apoptosis, we evaluated the apoptotic effects of TMS-TMF-4f on STAT3-overexpressing cervical cancer cells with pMXs-STAT3C transfection. As shown in Figure 3c,d, ectopically expressed STAT3 completely attenuated TMS-TMF-4f-induced apoptotic cell death and PARP cleavage in both cervical cancer cells compared with TMS-TMF-4f-treated control cells. These results suggest that STAT3 may be a crucial regulator during TMS-TMF-4f-induced apoptosis in cervical cancer cells.

### 2.4. TMS-TMF-4f Ameliorates IL-6-Induced STAT3 Activation in Human Cervical Cancer Cells

**Figure 4:**
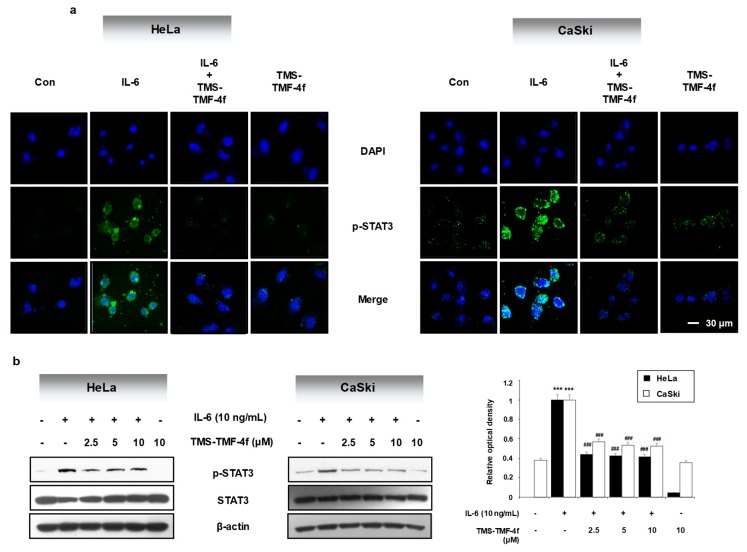
TMS-TMF-4f reduces IL-6-mediated STAT3 activation in human cervical cancer cells. After pretreatment with TMS-TMF-4f for 1 h, cells were stimulated with IL-6 (10 ng/mL) for 5 min, and STAT3 activation was determined by (**a**) immunofluorescence and (**b**) Western blot analysis. The relative optical density ratio was determined using a densitometric analysis program (Bio-Rad Quantity One^®^ Software, version 4.6.3 (Basic)), normalized to the internal control. Data are presented as the mean ± SD of three independent experiments. *** *p* < 0.001 vs. the control group. ### *p* < 0.001 vs. the TMS-TMF-4f-treated group.

Among the various stimulators of STAT3 activation, IL-6 is a key growth factor for tumorigenesis, and its production is considered as an important factor for linking inflammation to cancer [27]. To investigate the possible effects of TMS-TMF-4f on IL-6-induced STAT3 activation, we examined the effects of TMS-TMF-4f on IL-6-induced STAT3 phosphorylation. Pretreatment with TMS-TMF-4f reduced the green fluorescence intensity (IL-6-induced STAT3 phosphorylation) and p-STAT protein levels in both HeLa and CaSki cervical cancer cells (Figure 4a,b).

### 2.5. TMS-TMF-4f Docks into a Pocket Between the DNA-Binding and Ligand-Binding Domains of STAT3

**Figure 5:**
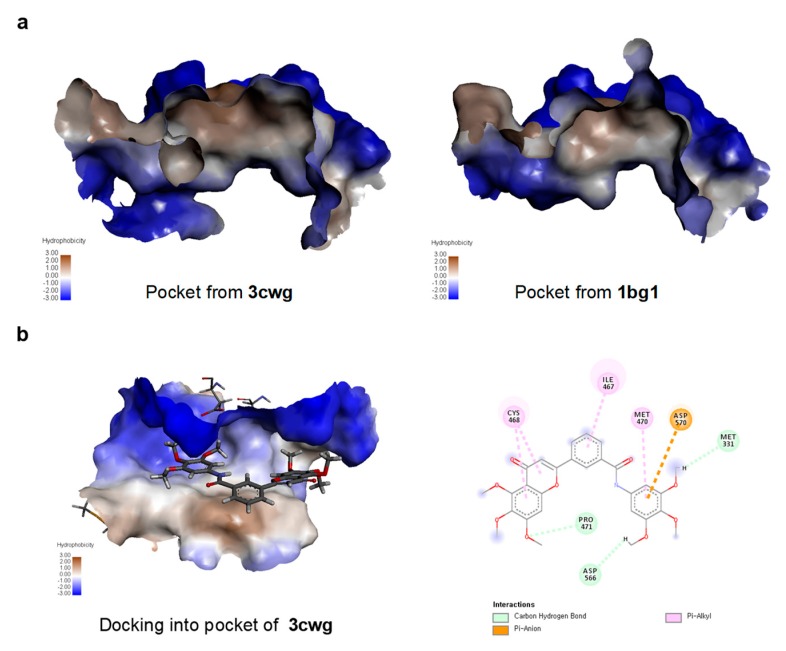
TMS-TMF-4f dock into unphosphorylated DNA-bound STAT3. (**a**) Binding of tectochrysin to phosphorylated DNA-bound STAT3 (1bg1) and unphosphorylated DNA-bound STAT3 (3cwg). (**b**) Docking model of TMS-TMF-4f with STAT3.

To gain insights into the underlying molecular interactions that mediate the inhibition of STAT3 activation by TMS-TMF-4f, a docking study was conducted. Among the few available crystal structures of STAT3 in the Protein Databank, STAT3 co-crystallized with the inhibitor compound tectochrysin (PDB code: 3cwg) showed that tectochrysin binding to a hydrophobic pocket between the DNA-binding domain (DBD) and ligand-binding domain (LBD) of unphosphorylated STAT3 may be responsible for the inhibition of phosphorylation and DNA binding with STAT3 [28]. Interestingly, the size and shape of the tectochrysin pocket differed from the crystal structure of phosphorylated DNA-bound STAT3 (PDB code: 1bg1; Figure 5a). As the actual binding site was not known, the docking study used a blind docking approach with both crystals (3cwg and 1bg1). Analysis of the docking run demonstrated that TMS-TMF successfully docked into the tectochrysin pocket of the unphosphorylated STAT3 (3cwg) but not the phosphorylated DNA-bound STAT3 (Figure 5b). These results suggest that the binding of TMS-TMF to this pocket may inhibit the interconversion of STAT3 to the activated form, resulting in the growth inhibition of cervical cancer cells.

### 2.6. TMS-TMF-4f Exhibits Significant Antitumor Effects in a Xenograft Cervical Cancer Mouse Model

**Figure 6:**
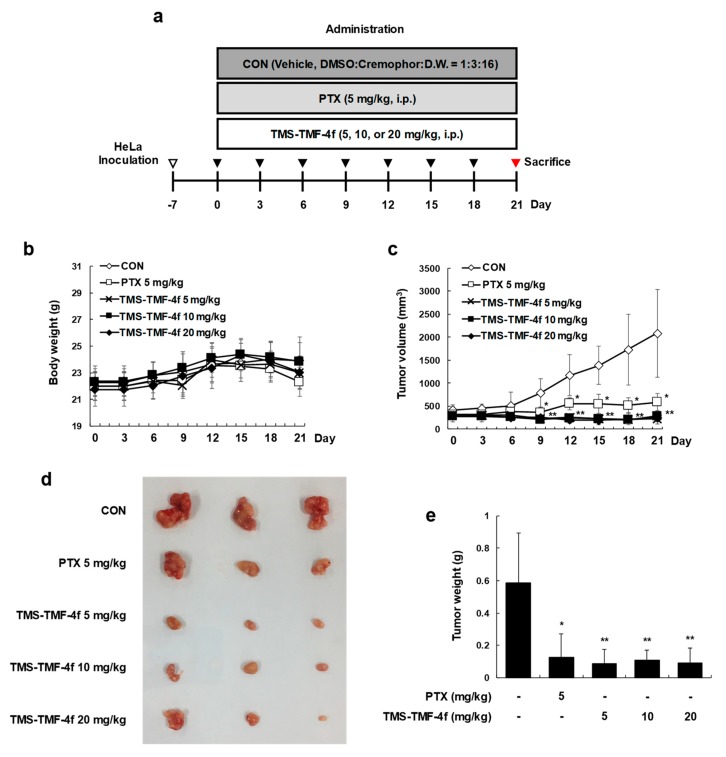
TMS-TMF-4f inhibits tumor growth in a HeLa xenograft model. (**a**) Schematic diagram of the in vivo experiment. HeLa cells were inoculated subcutaneously into BALB/c nude mice, and HeLa xenografts were treated intraperitoneally with the vehicle (once every 3 days), paclitaxel (5 mg/kg, once every 3 days), or TMS-TMF-4f (5, 10, or 20 mg/kg, once every 3 days) for 21 days. (**b**) Tumor volume (mm^3^) and (**c**) body weight (g) were measured throughout the experimental period. (**d**) The tumors were separated and (**e**) weighed after the mice were sacrificed. Data are presented as the mean ± SD (*n* = 8). * *p* < 0.05, ** *p* < 0.01 vs. the control group.

As the in vitro results were promising, we evaluated the ability of TMS-TMF-4f to inhibit the growth of human cervical cancer cells subcutaneously implanted in BALB/c nude mice. The experimental protocol is outlined in Figure 6a. One week after tumor cell injection, the animals were randomized into five treatment groups with tumor volumes of around 300 mm^3^ for each group. Treatment began 1 week after implantation and continued for up to 21 days. The animals were sacrificed at 21 days after the initiation of treatment, and the diameters of the excised tumors were measured. Although the total body weight of the paclitaxel-treated group was decreased compared with that of the vehicle-treated control group, no significant difference in the average total body weight was observed between the vehicle-treated control and TMS-TMF-4f-treated groups (Figure 6b). The intraperitoneal (i.p.) administration of TMS-TMF-4f (5, 10, or 20 mg/kg/day, once every three days for three weeks) or paclitaxel (5 mg/kg/day, once every three days for three weeks) significantly reduced the tumor volume compared with the volume in the vehicle-treated control group (Figure 6c,d). In addition, compared with the tumor weight in the vehicle-treated control group (0.588 ± 0.304 g), the tumor weight in the TMS-TMF-4f-treated groups was significantly reduced (5 mg/kg TMS-TMF-4f-treated group: 0.086 ± 0.088 g, *p* < 0.01; 10 mg/kg TMS-TMF-4f-treated group: 0.109 ± 0.063 g, *p* < 0.01; 20 mg/kg TMS-TMF-4f-treated group: 0.093 ± 0.092 g, *p* < 0.01) (Figure 6e).

### 2.7. TMS-TMF-4f Promotes Apoptosis and Attenuates STAT3 Activation in Cervical Tumor Tissues

**Figure 7:**
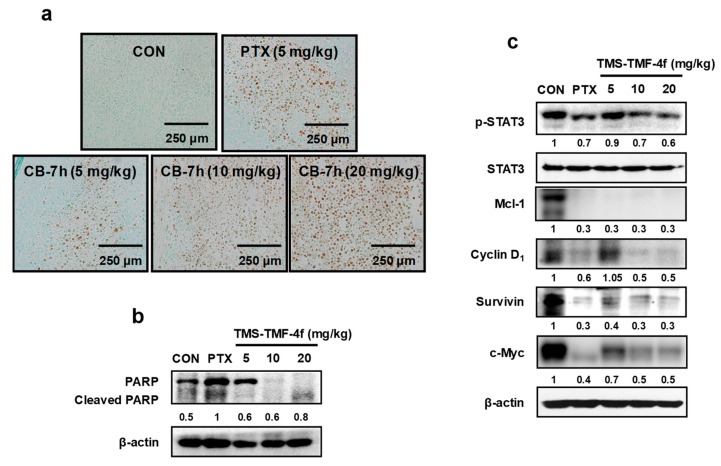
TMS-TMF-4f-induced apoptosis is associated with STAT3 inactivation in a HeLa xenograft model. (**a**) Apoptosis induction was examined by TUNEL assay using tumor sections (scale bar = 250 μm). (**b**,**c**) Tumor tissues were homogenized after 21 days of TMS-TMF-4f treatment, and whole proteins were prepared for western blotting to detect the protein expression of PARP, p-STAT3, STAT3, Mcl-1, cyclin D_1_, survivin, and c-Myc. β-actin was used as an internal control. The relative optical density ratio was determined using a densitometric analysis program (Bio-Rad Quantity One^®^ Software, version 4.6.3 (Basic), normalized to the internal control.

We also examined whether TMS-TMF-4f can modulate the induction of apoptosis and expression of various oncogenic gene products in tumor tissues. Treatment with TMS-TMF-4f enhanced the induction of apoptosis, as demonstrated by TUNEL assay and Western blot analysis of PARP cleavage in cervical tumor tissues (Figure 7a,b). In addition, consistent with the in vitro results for HeLa and CaSki cervical cancer cells, Western blot analysis indicated that treatment with TMS-TMF-4f inhibited the phosphorylation of STAT3 and its associated proteins, Mcl-1, cyclin D_1_, survivin, and c-Myc, in the tumor tissues of the xenograft mice (Figure 7c).

## 3. Discussion

In the present study, we describe for the first time the anti-cancer activity of TMS-TMF-4f in vitro and in vivo. Furthermore, we report that TMS-TMF-4f could inhibit the induction of proliferative mediators such as Mcl-1, cyclin D_1_, survivin, and c-Myc via STAT3 inactivation, subsequently inducing apoptotic cell death in human cervical cancer cells.

Although we previously reported the synthesis of TMS and TMF hybrid analogs and their growth inhibitory effects on various cancer cells [16], only percent growth inhibition values were determined. In this study, we examined the mechanism involved in the cytotoxicity of hybrid TMS-TMF compounds in human cervical carcinoma cells. Among the hybrid analogs, TMS-TMF-4f was observed to elicit broad and potent cytotoxic effects on human colorectal, lung, pancreatic, and cervical cancer cells. In particular, TMS-TMF-4f was the most effective against HeLa human cervical cancer cells and was more selective for cervical cancer cells than for normal ovarian epithelial cells (IOSE-80PC). In addition, xenograft assay showed that the effective dose of TMS-TMF-4f (5, 10, or 20 mg/kg, i.p.) had no apparent systemic toxicity in vivo, consistent with an earlier finding showing that TMS-T TMF-4f may be effective and safe for the treatment of cervical cancer.

To date, the mechanisms underlying the anti-cancer activity of TMS-TMF-4f remain unclear, and the cellular targets of TMS-TMF-4f in cervical cancer cells remain unidentified. In the present study, we found that the cytotoxic effects of TMS-TMF-4f may be attributed to apoptosis induction. Apoptosis is a cell death process, which is characterized by distinct morphological features and energy-dependent biochemical mechanisms [29]. The loss of apoptotic control allows cancer cells to survive longer and provides more time for the accumulation of mutations, which can increase invasiveness during tumor progression, stimulate angiogenesis, deregulate cell proliferation, and interfere with differentiation [30]. Recent advances in cancer research have focused on the development of new agents that can halt the escape behavior of cancer cells via apoptosis induction [31]. Indeed, targeting components of the apoptotic pathway as a therapeutic approach in cancer would be effective considering that aberrant apoptosis is central to tumor growth and resistance development to anti-cancer therapies [32]. The mechanisms of apoptosis include the intrinsic and extrinsic pathways, which can interact with each other and involve an energy-dependent cascade of molecular events [33]. The extrinsic pathway is initiated by transmembrane receptor-mediated interactions, which involve death receptors that are members of the tumor necrosis factor (TNF) receptor family [34]. Death receptors play a critical role in transmitting death signals from the cell surface to the intracellular signaling pathways [35]. We examined the effects of TMS-TMF-4f on extrinsic pathway proteins, such as Fas, FasL, and FADD, and found that the expression levels of these proteins were not affected by TMS-TMF-4f. On the other hand, the intrinsic pathways involve various non-receptor-mediated stimuli to produce intracellular signals that act directly on targets within the cell and are mitochondria-initiated events [30]. The regulation of mitochondrial events occurs through the Bcl-2 family anti-apoptotic proteins (Bcl-2 and Bcl-xL) and pro-apoptotic proteins (Bax, Bad, and Bid) [36]. The main mechanism of Bcl-2 family proteins involves the induction of the MMP, which leads to the release of cytochrome *c* and Smac/DIABLO from the mitochondria to cytosol [37]. The released pro-apoptotic proteins (cytochrome *c* and Smac/DIABLO) activate the caspase-dependent mitochondrial pathway [38]. In agreement with these findings, we found that TMS-TMF-4f treatment altered the expression levels of Bcl-2 family proteins and induced MMP loss, releasing cytochrome *c* and Smac/DIABLO into the cytosol, which contributed to apoptosis induction in HeLa and CaSki cervical cancer cells.

Several studies reported that constitutive and IL-6-induced STAT3 activation is common in cervical cancer cell lines and tissues [39,40]. Additionally, Shirish et al. demonstrated that STAT3 is aberrantly expressed and constitutively activated in cervical cancer, which increases as the lesion progresses, thus indicating its potential role in the progression of HPV16-mediated cervical carcinogenesis [41]. STAT3 phosphorylation at tyrosine 705 (Y705) is required for STAT3 dimerization and nuclear translocation. Dimeric STAT3 binds to specific DNA response elements in the promoters of target genes [42]. Therefore, STAT3-specific inhibitors may be useful for cervical cancer treatment, and various STAT3 inhibitors are in the process of being tested in clinical trials [41]. As expected, TMS-TMF-4f treatment suppressed STAT3 phosphorylation specifically at Y705 and downregulated the expression of proteins such as Mcl-1, cyclin D_1_, survivin, and c-Myc in HeLa and CaSki cells. Furthermore, the overexpression of exogenous STAT3 markedly reduced TMS-TMF-4f-induced apoptotic cell death. Interestingly, in silico docking analysis of TMS-TMF interaction with STAT3 revealed that the binding of TMS-TMF to a hydrophobic pocket between the DBD and LBD of unphosphorylated STAT3 (3cwg) may inhibit its conversion to the active phosphorylated form. This was consistent with the result showing that TMS-TMF decreased the levels of p-STAT3 and STAT-3-regulated proteins. Given these findings, we believe that TMS-TMF might regulate an upstream STAT3 activator. Among over 40 different STAT3 activators, IL-6 is the most representative cytokine, which serves a critical function in STAT3 signaling and inhibits apoptosis in toxic environments during inflammation [43]. High levels of IL-6 have been detected in various types of human epithelial cancers and correlate with the proliferation or survival of cervical cancer cells [44]. Therefore, we hypothesized that IL-6 could be a key target in TMS-TMF-4f-suppressed STAT3 activation. As expected, our results demonstrated that IL-6-induced STAT3 activation was reduced in TMS-TMF-4f-treated cervical cancer cells.

To verify the in vivo relevance of the in vitro results showing the anti-proliferative effects of TMS-TMF-4f, we evaluated its effects in a BALB/c nude mouse xenograft model with subcutaneous HeLa xenografts. Treatment with TMS-TMF-4f reduced tumor volumes and tumor weights. Consistent with in vitro findings, TMS-TMF-4f-induced DNA fragmentation and PARP cleavage were detected by TUNEL assay and Western blot analysis, respectively, in the tumor tissues of TMS-TMF-4f-treated HeLa xenograft mice, indicating that TMS-TMF-4f-induced apoptosis may be a critical mechanism against cervical tumor progression. In addition, TMS-TMF-4f effectively reduced the protein levels of p-STAT3 and STAT3-regulated oncogenic targets (the anti-apoptotic proteins Mcl-1 and survivin, the cell cycle regulatory protein cyclin D_1_, and the oncoprotein c-Myc). Taken together, targeting the STAT3 signaling pathway using TMS-TMF-4f could be a potential approach in human cervical cancer therapy.

## 4. Materials and Methods

### 4.1. Synthesis of Hybrid Analogs

Compounds used in this study were synthesized as previously reported [16].

### 4.2. Cell Culture

HeLa (human cervix adenocarcinoma) was cultured in DMEM added with 10% fetal bovine serum and 1% penicillin-streptomycin (Life Technologies Inc., Grand Island, NY, USA). HCT116 (human colorectal carcinoma), A549 (human lung carcinoma), AsPC-1 (human pancreatic adenocarcinoma), and CaSki (human cervix epidermoid carcinoma) and IOSE-80PC (human ovarian epithelial cell) were cultured in RPMI 1640, which also added FBS and penicillin-streptomycin. All cells were purchased from Korea cell line bank (Seoul, Korea) and grown at 37 °C under 5% CO_2_.

### 4.3. Cell Viability Assay

Cells were seeded (1 × 10^5^/mL) and incubated with various concentrations (0, 1.56, 3.13, 6.25, 12.5, and 25 μM) of six hybrid analogs and etoposide for 48 h. Then, 20 μL of MTT solution (5 mg/mL in PBS) was added and the cells were reacted for 4 h. The medium was aspirated and purple formazan crystals were dissolved with 200 μL of DMSO for 24 h with shaking. The absorbance was detected by micromultiplate reader at 540 nm.

### 4.4. Annexin V-FITC and PI double Staining Assay

Cells were seeded (1 × 10^5^/mL) and incubated with various concentration (0, 2.5, 5, and 10 μM) of TMS-TMF-4f for 48 h. After treatment with TMS-TMF-4f, Annexin V-FITC and PI double staining was performed by Annexin V-FITC Apoptosis Detection kit I (BD Bioscience Pharmingen, CA, USA), following the manufacturer’s instruction and analyzed by a flow cytometer, Cytomics FC 500 (Beckman Coulter, CA, USA).

### 4.5. Western Blot Analysis

Cells and tumor tissues were harvested and lysed in protein lysis buffer (Intron, Seoul, Korea) for 30 min at 4 °C. Cell debris was removed by microcentrifugation (4 °C, 15,000 rpm, 30 min) and then the protein concentration of supernatants was ascertained by Bio-rad protein assay reagent, following the manufacturer’s instruction. Cell extract was fractionated by 8–15% SDS PAGE and transferred to a PVDF. After blocking with 5% skim milk in Tween 20/Tris-buffered saline (T/TBS) for 30 min at 25 °C, the membrane was reacted with primary antibodies for 18 h at 4 °C. Membranes were washed three times with T/TBS for 10 min and incubated with secondary antibody for 2 h at 25 °C. And membranes were washed three times with T/TBS for 10 min and blots were developed using enhanced chemiluminescence detection agents (Amersham, Buckinghamshire, England). All original datea of Western blot are available in Supplemetnary File S1.

### 4.6. Measurement of MMP

Cells were seeded (1 × 10^5^/mL) and incubated with 10 µM TMS-TMF-4f for 12, 24, and 48 h. After treatment with TMS-TMF-4f, the cells were stained with 30 nM of DiOC_6_ for 30 min at 37 °C in dark. The stained cells were harvested, and the fluorescence intensity was determined with a flow cytometer, Cytomics FC 500 (Beckman Coulter, CA, USA).

### 4.7. Preparation of Cytosolic Fraction

Cells were seeded (1 × 10^5^/mL) and incubated with 10 µM TMS-TMF-4f for 12 and 48 h. To determine the release of cytochrome *c* and Smac/DIABLO from mitochondria to cytosol, cytosolic fractions from HeLa and CaSki cells were isolated by using the Mitochondria Isolation Kit, which is purchased from Pierce Biotechnology (Waltham, Massachusetts, USA). The isolation process followed the manufacturer’s instructions.

### 4.8. Immunocytochemistry

After co-treatment with TMS-TMF-4f and IL-6, cells were washed with PBS and then were fixed with 4% paraformaldehyde in PBS at 4 °C. Next day, to induce the cell permeability, cells were washed with PBS 3 times and were incubated with 0.3% Triton-X 100/PBS for 1 h at 25 °C. Cells were blocked with 10% normal goat serum in 0.3% Triton-X 100/PBS for 1 h and cells were incubated with p-STAT3 antibody in 5% normal goat serum in 0.3% Triton-X 100/PBS (dilution ration 1:200) at 4 °C. The following day, cells were washed with 0.3% Triton-X 100/PBS for 10 min 3 times and were incubated with Alexa Fluor 488-conjugated secondary antibody in 5% normal goat serum in 0.3% Triton-X 100/PBS (dilution ration 1:100) for 2 h at 25 °C in dark before counterstaining with 4′, 6-diamidino-2-phenylindole (DAPI). After glycerol mounting, images were captured using the K1-Fluo laser scanning confocal microscope (NANOSCOPE systems, Seoul, Korea).

### 4.9. Transfection

Cells were transfected with 12 μg of plasmid DNA (pMXs-gw and pMXs-STAT3C) by Lipofectamine^TM^ LTX (Invitrogen, Carlsbad, CA, USA) according to the manufacturer’s instruction and then incubated for 24 h prior to the treatment of TMS-TMF-4f.

### 4.10. Animals

The female BALB/c nude mice (6-week-old, 20–23 g) were obtained from Nara Biotec Co. (Pyeongtaek, Republic of Korea). Mice were inhabited 8/cage and were had standard laboratory chow in an animal room with 12 h dark/light cycles at a constant temperature of 20 ± 5 °C. All animal experiments were performed under university guidelines and were approved by the ethical committee for Animal Care and Use of Kyung Hee University according to the animal protocol (KHUASP(SE)-18-121).

### 4.11. In Vivo Tumor Xenograft Studies

HeLa cells (1 × 10^6^ per site) were inoculated subcutaneously into the right side of the flank of female BALB/c nude mice. Tumor size was checked with a caliper once per 3 days and calculated as *V* = π/6 × (length) × (width)^2^ [45]. When tumor volume reached around 300 mm^3^, mice were divided into 5 groups and treated with vehicle (DMSO: Cremophor: D.W. = 1:3:16, i.p.), paclitaxel (positive control, 5 mg/kg, i.p.) and TMS-TMF-4f (5, 10, or 20 mg/kg, i.p.) (Figure 5a). During the treatment, tumor volume and body weight were measured once per 3 days. On day 21, mice were killed and tumors were obtained.

### 4.12. Terminal Deoxynucleotidyl Transferase-Mediated dUTP μM Nick End Labeling (TUNEL) Assay

Tumor tissues were fixed 10% paraformaldehyde and embedded in paraffin. For TUNEL assay, tissue samples were sectioned (5 μm) and reacted TUNEL mixture according to the manufacturer’s instruction (in situ cell death detection kit, POD, Roche, Germany). The stained slides were observed with an Olympus microscope (Olympus cellSens Standard 1.9., Tokyo, Japan) and photographed.

### 4.13. Molecular Docking Analysis

The ligand was sketched, hydrogens added, energy minimized and saved as mol2 files. SwissDock which is based on the docking software EADock DSS running on the Vital-IT cluster was used to dock TMS-TMF-4f to the crystal structures (PDB codes: 3cwg and 1bg1, indicating unphosphorylated STAT3 and phosphorylated DNA-bound STAT3, respectively) in accurate mode as a blind docking study [46,47,48].

### 4.14. Statistical Analysis

Data are represented as the mean ± SD. of triplicate experiments. Statistical significances were identified using ANOVA and Dunnett’s post hoc test, and *P*-values of less than 0.05 were reputed statistically significant.

## 5. Conclusions

The current study clearly demonstrates that a novel compound, TMS-TMF-4f, which has low toxicity but is highly effective for treating human cervical cancer. TMS-TMF-4f treatment could not only induce cell death in cervical cancer cells but also prevent tumor growth in a xenograft cervical cancer model by suppressing STAT3 phosphorylation via the induction of apoptosis. Therefore, TMS-TMF-4f may be used as a potential agent in antitumor therapy for patients with cervical cancer. 

## Figures and Tables

**Table 1:** Chemical structure and IC_50_ value of modified hybrid compounds for inhibiting various cancer cell lines.

Compound (TMS-TMF)		^a^ IC_50_ (μM)			
		HCT116	A549	AsPC-1	HeLa
**4a**	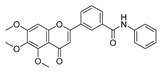	38.27 ± 1.60	46.61 ± 3.12	39.11 ± 2.23	35.55 ± 2.54
**4b**	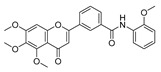	27.60 ± 7.55	27.86 ± 6.47	25.57 ± 6.48	14.21 ± 4.63
**4c**	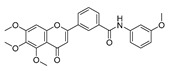	36.27 ± 4.76	24.41 ± 2.49	31.41 ± 1.45	34.99 ± 1.70
**4d**	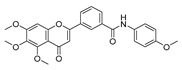	33.57 ± 5.38	41.85 ± 3.69	>50	>50
**4e**	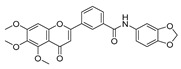	30.54 ± 5.21	>50	>50	>50
**4f**	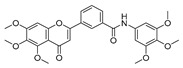	17.33 ± 2.76	16.79 ± 3.85	14.68 ± 1.65	12.07 ± 1.84
Etoposide		21.45 ± 3.82	24.25 ± 2.99	29.77 ± 3.08	32.70 ± 2.56

^a^ IC_50_ is the concentration that results in 50% reduction in cell viability relative to the control. Values are the mean of a triplicate assay.

## Supplementary Materials

The following are available online at https://www.mdpi.com/2072-6694/11/12/1927/s1, File S1, original data of western blot.
