# Supplementary material for: The Anti-Proliferative Activity of the Hybrid TMS-TMF-4f Compound Against Human Cervical Cancer Involves Apoptosis Mediated by STAT3 Inactivation
Source: Cancers (Basel). 2019 Dec 3;11(12):1927. doi: 10.3390/cancers11121927 (PMC6966466; doi:10.3390/cancers11121927)
Supplement: Supplementary file 1 [file cancers-11-01927-s001.pdf]

Figure 1c - HeLa

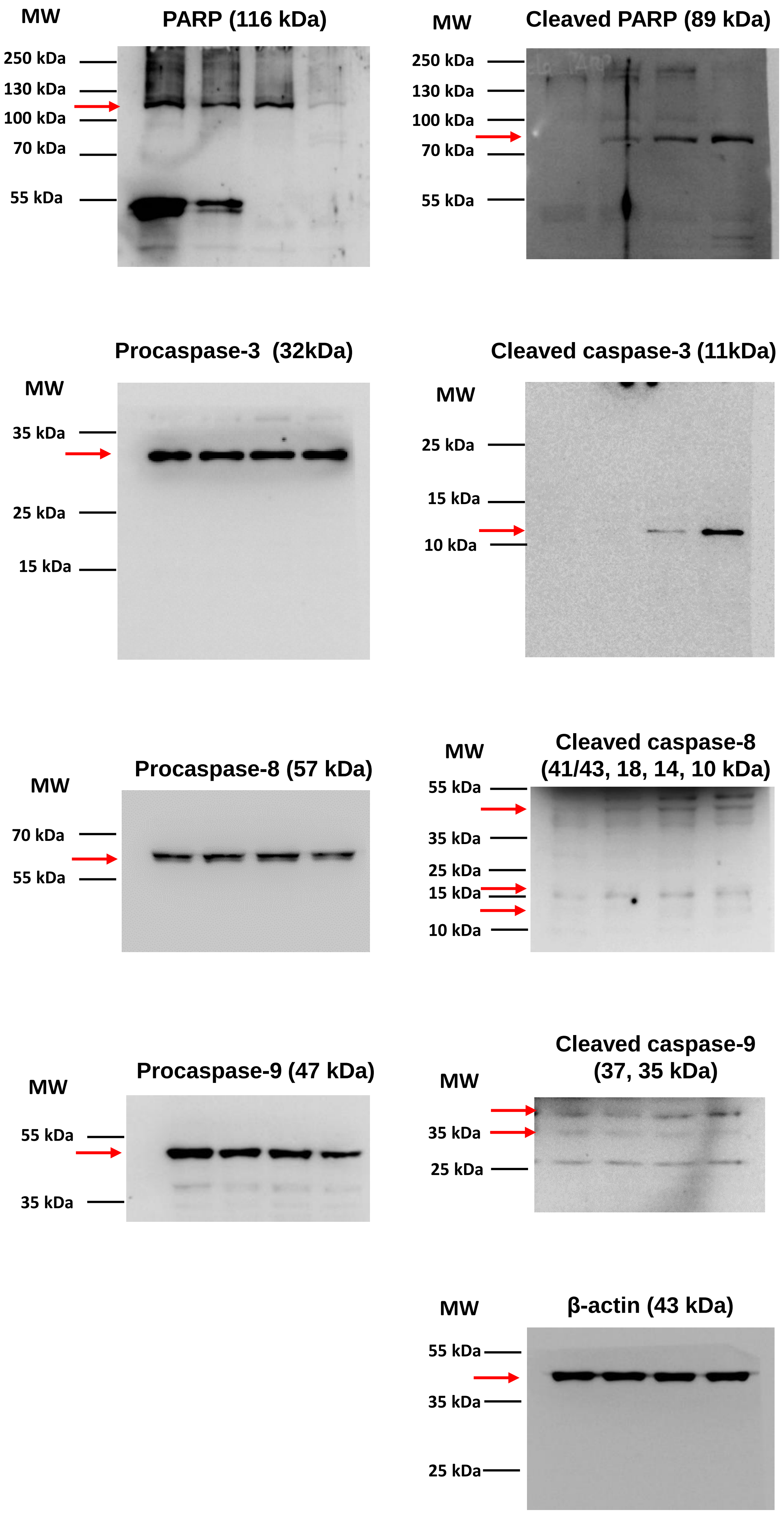

Fig.1.c-HeLa							
Gel name : TMS-TMF-4f _HeLa c PARP.tif (Raw 1-D Image)							
Index	Name	Volume	Adj. Vol.	% Adj. Vo	Concentration		
		INT*mm2	INT*mm2				
	(uM)					cleaved PARP/actin	Ratio
1	0	1246.507	1246.507	14.03441	N/A	0.213786754	0.456834
2	2.5	1604.642	1604.642	18.06665	N/A	0.272860504	0.583067
3	5	2847.794	2847.794	32.06329	N/A	0.421141266	0.899924
4	10	3182.847	3182.847	35.83565	N/A	0.467974328	1
Gel name : TMS-TMF-4f _Hela cleaved cas3 (Raw 1-D Image)							
Index	Name	Volume	Adj. Vol.	% Adj. Vo	Concentration		
		INT*mm2	INT*mm2				
	(uM)					cleaved cas3/actin	Ratio
1	0	379.3541	379.3541	7.10164	N/A	0.058967669	0.107687
2	2.5	382.9957	382.9957	7.169811	N/A	0.059128618	0.107981
3	5	1060.844	1060.844	19.85936	N/A	0.16578371	0.302756
4	10	3518.588	3518.588	65.86919	N/A	0.547581716	1
Gel name : TMS-TMF-4f _Hela cleaved cas8 (Raw 1-D Image)							
Index	Name	Volume	Adj. Vol.	% Adj. Vo	Concentration		
		INT*mm2	INT*mm2				
	(uM)					cleaved cas8/actin	Ratio
1	0	270.9099	270.9099	13.74195	N/A	0.043107279	0.477313
2	2.5	536.5724	536.5724	27.21772	N/A	0.085832891	0.950399
3	5	599.196	599.196	30.39431	N/A	0.099306909	1.099593
4	10	564.7301	564.7301	28.64602	N/A	0.090312459	1
Gel name : TMS-TMF-4f _Hela cleaved cas9 (Raw 1-D Image)							
Index	Name	Volume	Adj. Vol.	% Adj. Vo	Concentration		
		INT*mm2	INT*mm2				
	(uM)					cleaved cas9/actin	Ratio
1	0	230.7548	240.7451	20.34802	N/A	0.036186789	0.533931
2	2.5	230.0784	230.0784	19.44646	N/A	0.034813022	0.513662
3	5	274.2647	274.2647	23.18114	N/A	0.040418945	0.596376
4	10	438.0493	438.0493	37.02438	N/A	0.067774229	1
Gel name : TMS-TMF-4f _Hela bactin (Raw 1-D Image)							
Index	Name	Volume	Adj. Vol.	% Adj. Vo	Concentration		
		INT*mm2	INT*mm2				
	(uM)						
1	0	6984.904	6984.904	25.27511	N/A		
2	2.5	6999.212	6999.212	25.32689	N/A		
3	5	6832.732	6832.732	24.72447	N/A		
4	10	6818.653	6818.653	24.67353	N/A		

Figure 1c - CaSki

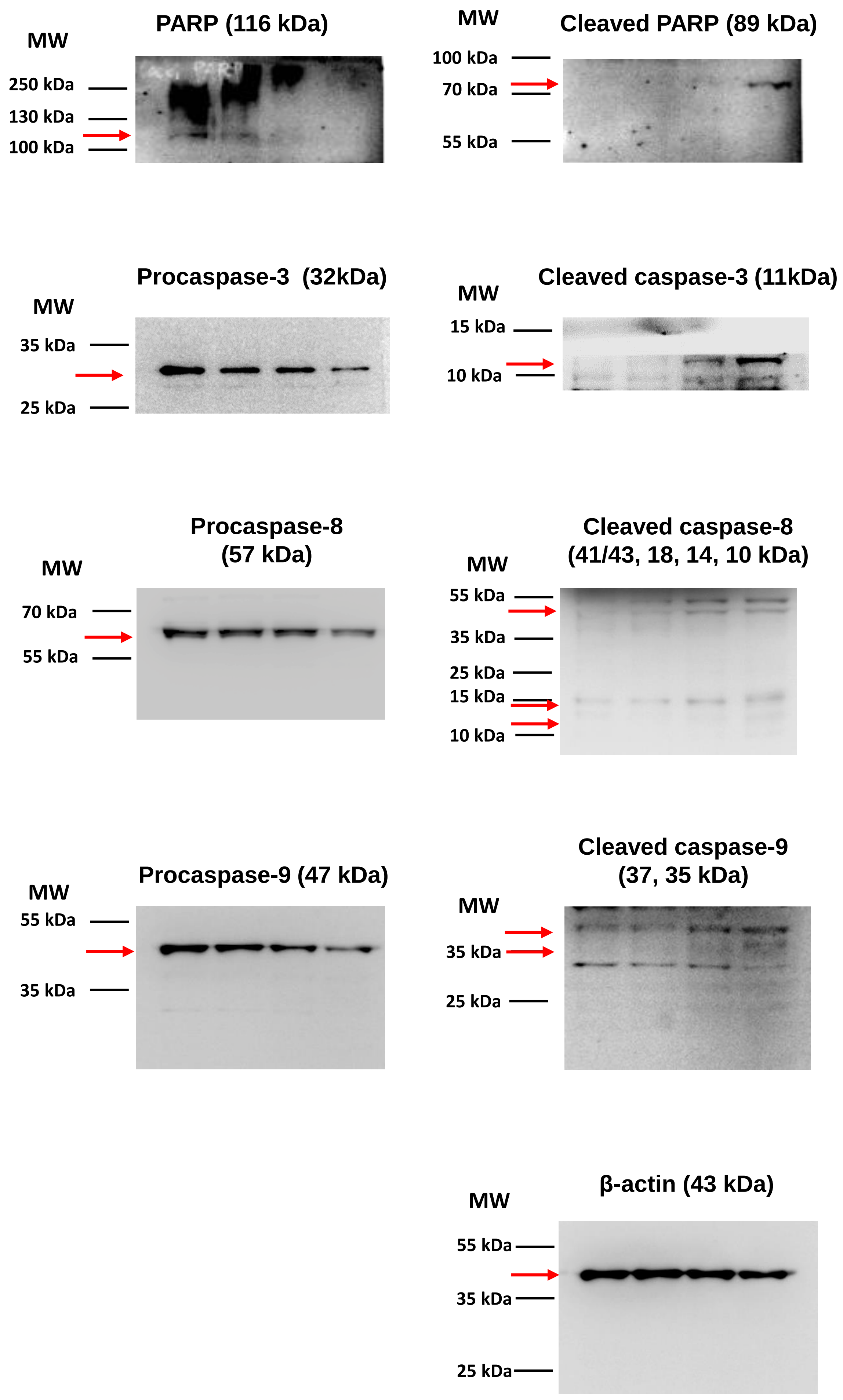

Fig.1.c-CaSki							
Gel name : TMS-TMF-4f_Caski c PARP.tif (Raw 1-D Image)							
**Index**	Name	Volume	Adj. Vol.	% Adj. Vo	Concentration		
		INT*mm2	INT*mm2				
	(uM)					cleaved PARP/actin	Ratio
1	0	2094.505	2094.505	19.74003	N/A	0.355073229	0.534769
2	2.5	2761.772	2761.772	26.02881	N/A	0.453268845	0.68266
3	5	2245.128	2245.128	21.1596	N/A	0.396543814	0.597227
4	10	3509.04	3509.04	33.07156	N/A	0.663974665	1
**Gel name : TMS-TMF-4f_caski cleaved cas3 v2 (Raw 1-D Image)**							
Index	Name	Volume	Adj. Vol.	% Adj. Vo	Concentration		
		INT*mm2	INT*mm2				
	(uM)					cleaved cas3/actin	Ratio
1	0	3345.054	3345.054	17.49902	N/A	0.540425606	0.46407
2	2.5	3804.58	3804.58	19.90295	N/A	0.617250495	0.530041
3	5	5686.641	5686.641	29.7486	N/A	0.980044659	0.841576
4	10	6279.386	6279.386	32.84943	N/A	1.164534456	1
**Gel name : TMS-TMF-4f_caski cleaved cas8 (Raw 1-D Image)**							
Index	Name	Volume	Adj. Vol.	% Adj. Vo	Concentration		
		INT*mm2	INT*mm2				
	(uM)					cleaved cas8/actin	Ratio
1	0	163.3258	163.3258	19.48283	N/A	0.028093089	0.511451
2	2.5	199.6842	199.6842	23.81995	N/A	0.034909359	0.635545
3	5	239.2827	239.2827	28.54358	N/A	0.044403525	0.808392
4	10	236.0139	236.0139	28.15365	N/A	0.054928196	1
**Gel name : TMS-TMF-4f_caski cleaved cas9 (Raw 1-D Image)**							
Index	Name	Volume	Adj. Vol.	% Adj. Vo	Concentration		
		INT*mm2	INT*mm2				
	(uM)					cleaved cas9/actin	Ratio
1	0	906.1488	906.1488	17.87605	N/A	0.162578924	0.474579
2	2.5	862.7367	862.7367	17.01964	N/A	0.149254058	0.435683
3	5	1538.606	1538.606	30.35286	N/A	0.282345441	0.824186
4	10	1761.574	1761.574	34.75145	N/A	0.342574975	1
**Gel name : TMS-TMF-4f_caski bactin (Raw 1-D Image)**							
Index	Name	Volume	Adj. Vol.	% Adj. Vo	Concentration		
		INT*mm2	INT*mm2				
	(uM)						
1	0	5317.036	5317.036	25.98747	N/A		
2	2.5	5513.681	5513.681	26.94858	N/A		
3	5	5108.721	5108.721	24.96931	N/A		
4	10	4520.565	4520.565	22.09464	N/A		

Figure 2a - HeLa

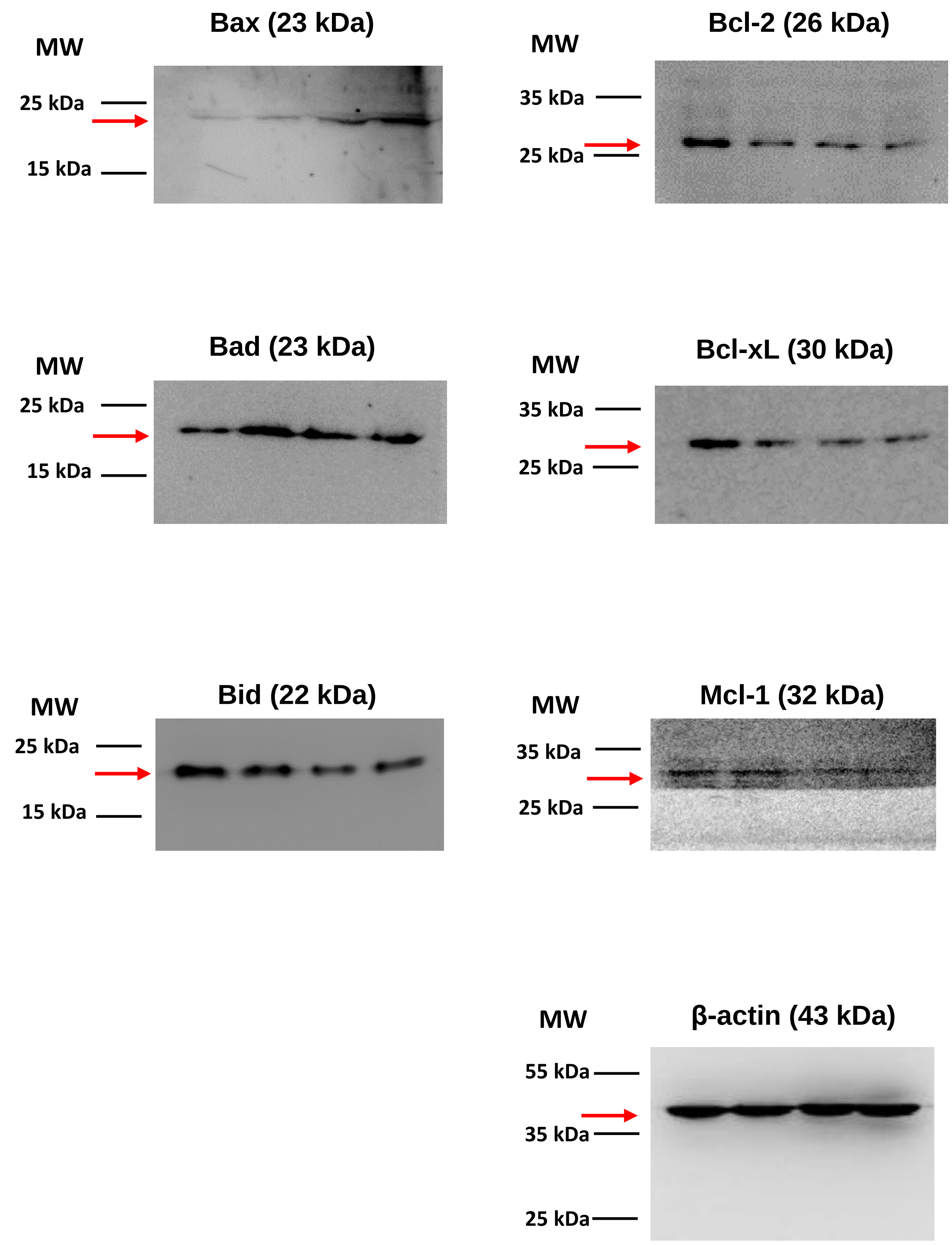

Fig.2.a-HeLa							
Gel name : TMS-TMF-4f _HeLa Bax (Raw 1-D Image)							
Index	Name	Volume	Adj. Vol.	% Adj. Vo	Concentration		
		INT*mm2	INT*mm2				
	(uM)					Bax/actin	Ratio
1	0	420.6443	420.6443	7.849663	N/A	0.09365207	0.220302
2	2.5	440.716	440.716	8.224221	N/A	0.094652643	0.222656
3	5	1291.754	1291.754	24.10548	N/A	0.335265816	0.788662
4	10	1205.643	3205.643	59.82064	N/A	0.425107222	1
Gel name :TMS-TMF-4f _HeLa Bad (Raw 1-D Image)							
Index	Name	Volume	Adj. Vol.	% Adj. Vo	Concentration		
		INT*mm2	INT*mm2				
	(uM)					Bad/actin	Ratio
1	0	1587.409	1587.409	15.00977	N/A	0.367821363	0.457216
2	2.5	3205.685	3305.685	31.25696	N/A	0.678614531	0.843543
3	5	2781.758	2781.758	26.30296	N/A	0.663552113	0.82482
4	10	2900.984	2900.984	27.4303	N/A	0.804480936	1
Gel name : TMS-TMF-4f _HeLa Bid (Raw 1-D Image)							
Index	Name	Volume	Adj. Vol.	% Adj. Vo	Concentration		
		INT*mm2	INT*mm2				
	(uM)					Bid/actin	Ratio
1	0	2711.679	2711.679	43.50266	N/A	0.616544605	1
2	2.5	1829.444	1829.444	29.34923	N/A	0.384188763	0.623132
3	5	750.2926	650.2926	10.43245	N/A	0.180897269	0.293405
4	10	741.9478	1041.948	16.71566	N/A	0.212667602	0.344935
Gel name : TMS-TMF-4f _HeLa Bcl-2 (Raw 1-D Image)							
Index	Name	Volume	Adj. Vol.	% Adj. Vo	Concentration		
		INT*mm2	INT*mm2				
	(uM)					Bcl-2/actin	Ratio
1	0	2434.805	2434.805	49.33963	N/A	0.549801152	1
2	2.5	941.0734	941.0734	19.0702	N/A	0.195186575	0.355013
3	5	940.6147	940.6147	19.0609	N/A	0.221883756	0.403571
4	10	618.2927	618.2927	12.52927	N/A	0.176662201	0.32132
Gel name : TMS-TMF-4f _HeLa Bcl Xl (Raw 1-D Image)							
Index	Name	Volume	Adj. Vol.	% Adj. Vo	Concentration		
		INT*mm2	INT*mm2				
	(uM)					Bcl-xL/actin	Ratio
1	0	2771.407	2771.407	46.97092	N/A	0.617261762	1
2	2.5	1217.345	1217.345	20.63206	N/A	0.243445272	0.394396
3	5	981.9336	981.9336	16.64221	N/A	0.222448293	0.360379
4	10	929.5753	929.5753	15.75482	N/A	0.253516998	0.410712
Gel name : TMS-TMF-4f _hela mcl-1 (Raw 1-D Image)							
Index	Name	Volume	Adj. Vol.	% Adj. Vo	Concentration		
		INT*mm2	INT*mm2				
	(uM)					Mcl-1/actin	Ratio
1	0	2388.239	2388.239	26.62026	N/A	0.728666776	1
2	2.5	2370.06	2370.06	26.41763	N/A	0.70805205	0.91288
3	5	2061.358	2061.358	22.97671	N/A	0.642737881	0.882074
4	10	2151.852	2151.852	23.9854	N/A	0.62633588	0.859564
Gel name : TMS-TMF-4f _HeLa Bcl2 fam bactin (Raw 1-D Image)							
Index	Name	Volume	Adj. Vol.	% Adj. Vo	Concentration		
		INT*mm2	INT*mm2				
	(uM)						
1	0	4373.497	4373.497	26.44293	N/A		
2	2.5	4689.625	4689.625	28.3543	N/A		
3	5	4085.182	4085.182	24.69973	N/A		
4	10	3391.076	3391.076	20.50304	N/A		

Figure 2a - CaSki

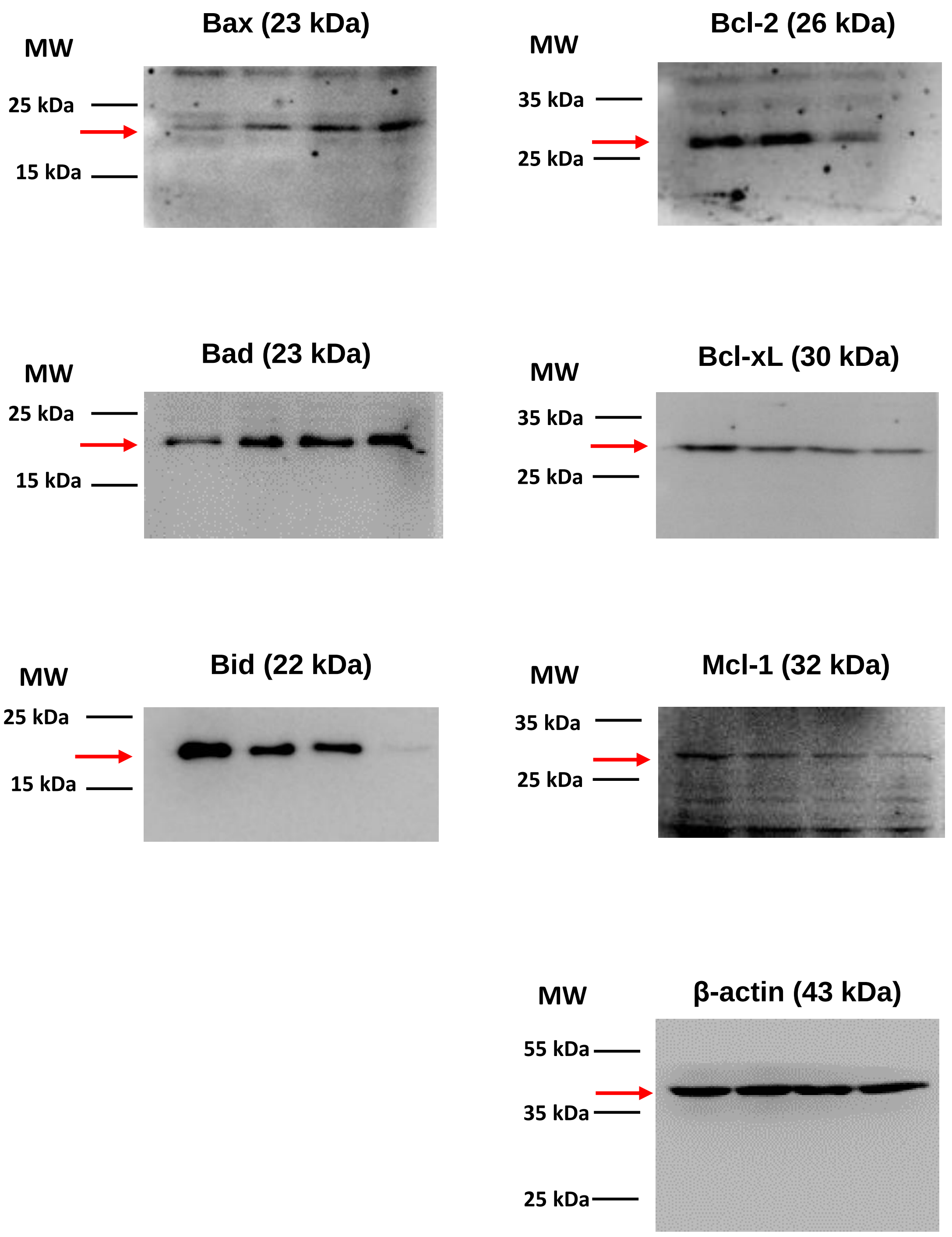

Fig.2.a-CaSki							
Gel name : TMS-TMF-4f _Caski Bax (Raw 1-D Image)							
Index	Name	Volume	Adj. Vol.	% Adj. Vo	Concentration		
		INT*mm2	INT*mm2				
	(uM)					Bax/actin	Ratio
1	0	239.1393	239.1393	4.789525	N/A	0.074053383	0.077633
2	2.5	707.2101	707.2101	14.16413	N/A	0.251850997	0.264026
3	5	1641.947	1641.947	32.8852	N/A	0.542587131	0.568816
4	10	2404.669	2404.669	48.16114	N/A	0.953888406	1
Gel name : TMS-TMF-4f _CaSki Bad (Raw 1-D Image)							
Index	Name	Volume	Adj. Vol.	% Adj. Vo	Concentration		
		INT*mm2	INT*mm2				
	(uM)					Bad/actin	Ratio
1	0	1139.238	1139.238	11.7019	N/A	0.368884284	0.273061
2	2.5	2439.823	2439.823	25.06111	N/A	0.86617171	0.641171
3	5	2819.435	2819.435	28.96037	N/A	0.932783726	0.690479
4	10	3336.997	3336.997	34.27661	N/A	1.350922001	1
Gel name : TMS-TMF-4f _CaSki Bid (Raw 1-D Image)							
Index	Name	Volume	Adj. Vol.	% Adj. Vo	Concentration		
		INT*mm2	INT*mm2				
	(uM)					Bid/actin	Ratio
1	0	4383.619	4383.619	50.73676	N/A	1.431648793	1
2	2.5	2219.895	2219.895	25.69345	N/A	0.78809729	0.550482
3	5	2036.412	2036.412	23.56978	N/A	0.676429227	0.472483
4	10	0	0	0	N/A	0	0
Gel name : TMS-TMF-4f _CaSki Bcl-2 (Raw 1-D Image)							
Index	Name	Volume	Adj. Vol.	% Adj. Vo	Concentration		
		INT*mm2	INT*mm2				
	(uM)					Bcl-2/actin	Ratio
1	0	3480.767	3480.767	46.05124	N/A	1.120519719	1
2	2.5	3216.194	3216.194	42.55089	N/A	1.11224746	0.992617
3	5	820.8443	820.8443	10.85993	N/A	0.268779384	0.23987
4	10	40.65942	40.65942	0.537932	N/A	0.016262915	0.014514
Gel name : TMS-TMF-4f _CaSki Bclxl (Raw 1-D Image)							
Index	Name	Volume	Adj. Vol.	% Adj. Vo	Concentration		
		INT*mm2	INT*mm2				
	(uM)					Bcl-xL/actin	Ratio
1	0	2259.809	2259.809	48.79576	N/A	0.752771501	1
2	2.5	1092.614	1092.614	23.59267	N/A	0.414477479	0.550602
3	5	1082.435	1082.435	23.37288	N/A	0.361023196	0.479592
4	10	196.3007	196.3007	4.238694	N/A	0.07966828	0.105833
Gel name : TMS-TMF-4f _caski mcl-1 (Raw 1-D Image)							
Index	Name	Volume	Adj. Vol.	% Adj. Vo	Concentration		
		INT*mm2	INT*mm2				
	(uM)					Mcl-1/actin	Ratio
1	0	2666.748	2666.748	38.57685	N/A	0.71497565	1
2	2.5	2018.491	2018.491	29.19925	N/A	0.479967517	0.671306
3	5	1485.015	1485.015	21.48205	N/A	0.377944331	0.528611
4	10	742.5649	742.5649	10.74185	N/A	0.2010054	0.281136
Gel name : TMS-TMF-4f _CaSki bcl2 fam bactin (Raw 1-D Image)							
Index	Name	Volume	Adj. Vol.	% Adj. Vo	Concentration		
		INT*mm2	INT*mm2				
	(uM)						
1	0	3061.614	3061.614	26.9949	N/A		
2	2.5	2795.808	2795.808	24.65123	N/A		
3	5	3012.037	3012.037	26.55777	N/A		
4	10	2471.995	2471.995	21.7961	N/A		

Figure 2c

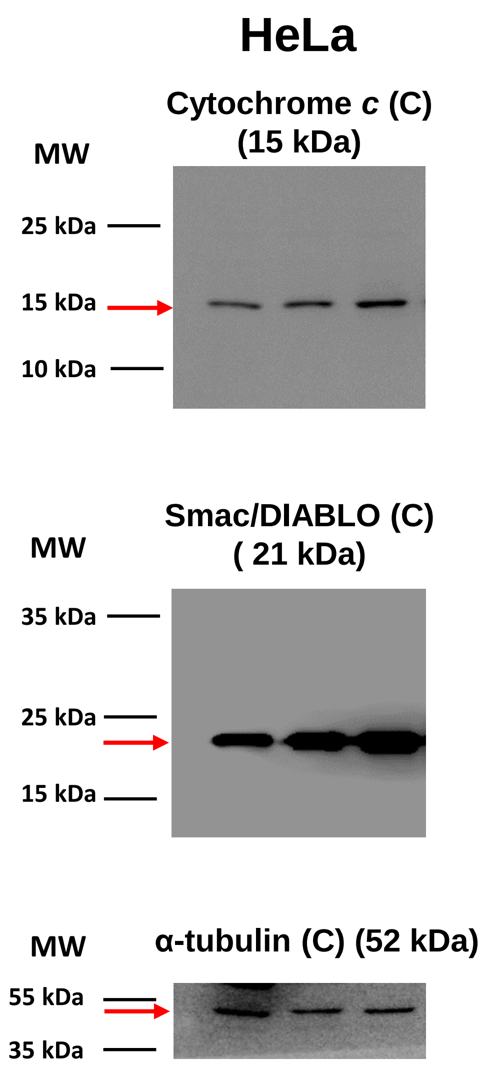

Fig.2.c-HeLa							
Gel name : TMS-TMF-4f _HeLa cytoC (Raw 1-D Image)							
Index	Name	Volume	Adj. Vol.	% Adj. Vo	Concentration		
		INT*mm2	INT*mm2				
	h					cytochrome c/tubulin	Ratio
1	0	535.8843	535.8843	20.04548	N/A	0.291522626	0.192273
2	12	771.8695	771.8695	28.87282	N/A	0.836695366	0.551841
3	24	1365.589	1365.589	51.0817	N/A	1.516188763	1
Gel name : TMS-TMF-4f _HeLa smac (Raw 1-D Image)							
Index	Name	Volume	Adj. Vol.	% Adj. Vo	Concentration		
		INT*mm2	INT*mm2				
	h					smac/tubulin	Ratio
1	0	3252.381	3252.381	22.05592	N/A	1.767007173	0.238529
2	12	4880.736	4880.736	33.09857	N/A	5.328878252	0.719346
3	24	6612.948	6612.948	44.84551	N/A	7.407946249	1
Gel name : TMS-TMF-4f _HeLa tubulin (Raw 1-D Image)							
Index	Name	Volume	Adj. Vol.	% Adj. Vo	Concentration		
		INT*mm2	INT*mm2				
	h						
1	0	1892.355	1892.355	50.42173	N/A		
2	12	949.1595	949.1595	25.29032	N/A		
3	24	911.5395	911.5395	24.28794	N/A		

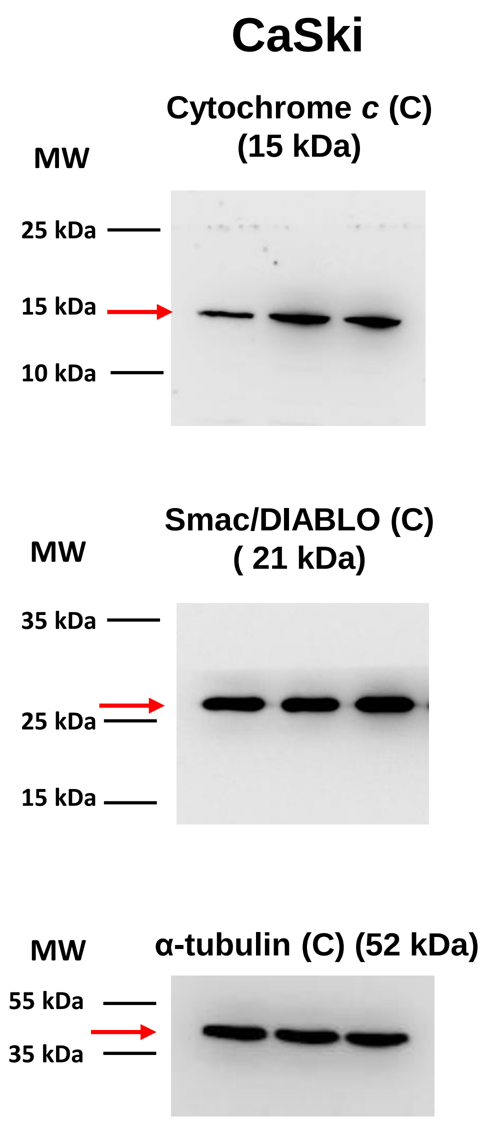

Fig.2.c-CaSki							
Gel name : TMS-TMF-4f _Caski cyto c.tif (Raw 1-D Image)							
Index	Name	Volume	Adj. Vol.	% Adj. Vo	Concentration		
		INT*mm2	INT*mm2				
	h					cytochrome c/tubulin	Ratio
1	0	1181.819	1181.819	18.75253	N/A	0.371166742	0.447989
2	12	2640.482	2640.482	41.89791	N/A	0.845990331	1.021089
3	24	2479.881	2479.881	39.34956	N/A	0.828517978	1
Gel name : TMS-TMF-4f _caski smac (Raw 1-D Image)							
Index	Name	Volume	Adj. Vol.	% Adj. Vo	Concentration		
		INT*mm2	INT*mm2				
	h					smac/tubulin	Ratio
1	0	3682.143	3682.143	31.28685	N/A	1.144623038	0.781234
2	12	3660.552	3660.552	31.10339	N/A	1.182337307	0.806975
3	24	4426.285	4426.285	37.60976	N/A	1.465147688	1
Gel name : TMS-TMF-4f _Caski bactin.tif (Raw 1-D Image)							
Index	Name	Volume	Adj. Vol.	% Adj. Vo	Concentration		
		INT*mm2	INT*mm2				
	h						
1	0	3285.27	3285.27	34.46705	N/A		
2	12	3197.04	3197.04	33.54141	N/A		
3	24	3049.313	3049.313	31.99154	N/A		

Figure 3a

HeLa

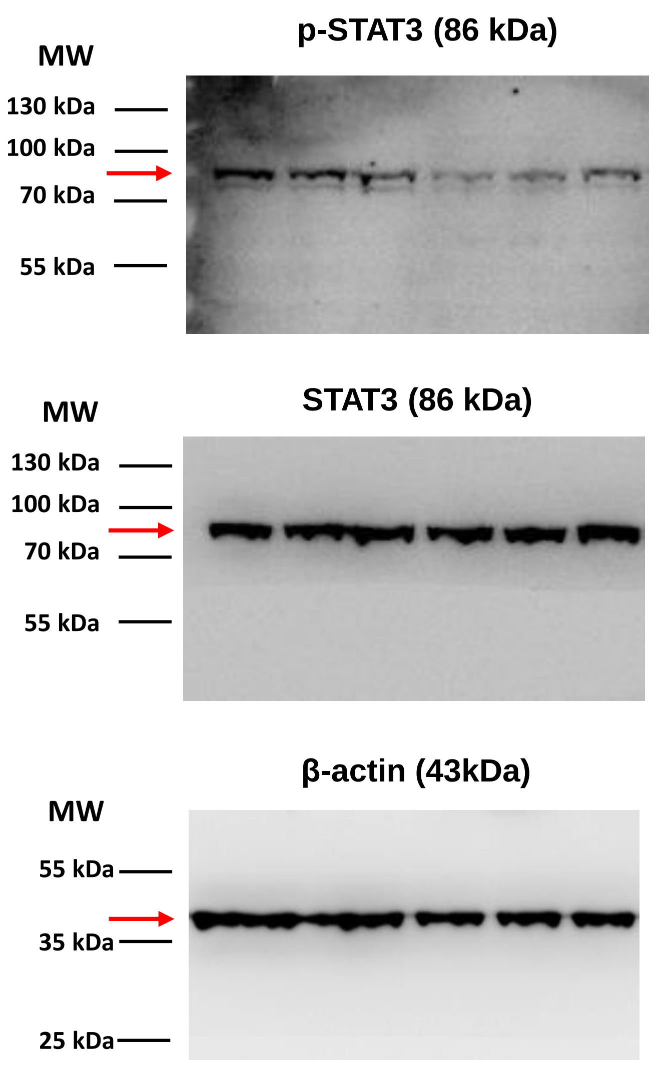

Fig.3.a-HeLa

Gel name : TMS-TMF-4f _HeLa pSTAT3 (Raw 1-D Image)

Index	Name	Volume	Adj. Vol.	% Adj. Vo	Concentration		
		INT*mm2	INT*mm2				
	min					p-stat3/stat3	Ratio
1	0	3016.424	3016.424	35.33929	N/A	0.799890149	1
2	5	1821.473	1821.473	21.33969	N/A	0.512225391	0.64037
3	10	1269.417	1269.417	14.87201	N/A	0.327827951	0.409841
4	15	671.2532	671.2532	7.864149	N/A	0.19211649	0.240179
5	30	749.5326	749.5326	8.781242	N/A	0.209141842	0.261463
6	60	1007.511	1007.511	11.80361	N/A	0.236063403	0.29512

Gel name : TMS-TMF-4f _HeLa STAT3 (Raw 1-D Image)

Index	Name	Volume	Adj. Vol.	% Adj. Vo	Concentration
		INT*mm2	INT*mm2		
	min				
1	0	3654.33	3654.33	16.12293	N/A
2	5	3513.57	3513.57	15.5019	N/A
3	10	3903.677	3903.677	17.22305	N/A
4	15	3607.161	3607.161	15.91482	N/A
5	30	3710.875	3710.875	16.37241	N/A
6	60	4275.805	4275.805	18.86489	N/A

Gel name : TMS-TMF-4f _HeLa bactin (Raw 1-D Image)

Index	Name	Volume	Adj. Vol.	% Adj. Vo	Concentration
		INT*mm2	INT*mm2		
	min				
1	0	4249.684	4249.684	18.39252	N/A
2	5	3736.71	3736.71	16.17238	N/A
3	10	4373.726	4373.726	18.92937	N/A
4	15	3510.818	3510.818	15.19472	N/A
5	30	3598.731	3598.731	15.57521	N/A
6	60	3635.835	3635.835	15.7358	N/A

CaSki

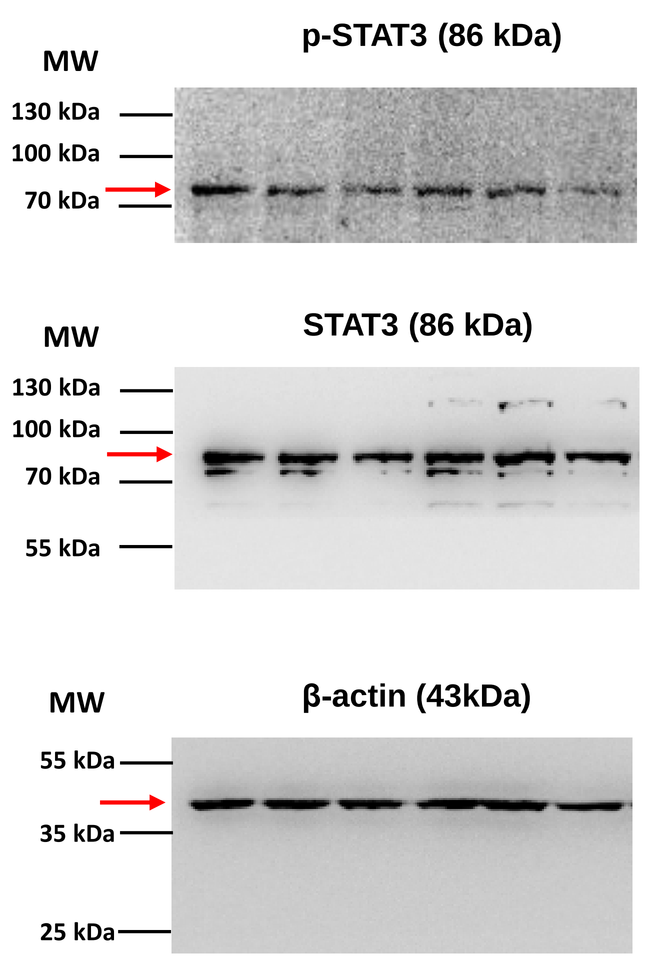

Fig.3.a-CaSki

Gel name : TMS-TMF-4f _Caski pSTAT3 (Raw 1-D Image)

Index	Name	Volume	Adj. Vol.	% Adj. Vo	Concentration		
		INT*mm2	INT*mm2				
	min					p-stat3/stat3	Ratio
1	0	2223.795	2223.795	24.4555	N/A	0.737024054	1
2	5	1404.556	1404.556	15.44618	N/A	0.534662055	0.725434
3	10	1167.854	1167.854	12.84312	N/A	0.527930481	0.7163
4	15	1637.416	1837.416	20.20642	N/A	0.556708883	0.755347
5	30	1569.775	1569.775	17.26312	N/A	0.494582594	0.671054
6	60	889.8334	889.8334	9.785669	N/A	0.34994909	0.474814

Gel name : TMS-TMF-4f _Caski stat3 (Raw 1-D Image)

Index	Name	Volume	Adj. Vol.	% Adj. Vo	Concentration
		INT*mm2	INT*mm2		
	min				
1	0	2939.378	2939.378	18.5095	N/A
2	5	2614.361	2614.361	16.46284	N/A
3	10	2141.214	2141.214	13.4834	N/A
4	15	2824.769	2824.769	17.7878	N/A
5	30	2997.786	2997.786	18.87731	N/A
6	60	2362.863	2362.863	14.87914	N/A

Gel name : TMS-TMF-4f _caski bactin (Raw 1-D Image)

Index	Name	Volume	Adj. Vol.	% Adj. Vo	Concentration
		INT*mm2	INT*mm2		
	min				
1	0	2282.318	2282.318	17.06926	N/A
2	5	2294.246	2294.246	17.15847	N/A
3	10	2093.043	2093.043	15.65368	N/A
4	15	2285.329	2285.329	17.09178	N/A
5	30	2562.031	2562.031	19.16121	N/A
6	60	1853.961	1853.961	13.86561	N/A

Figure 3b

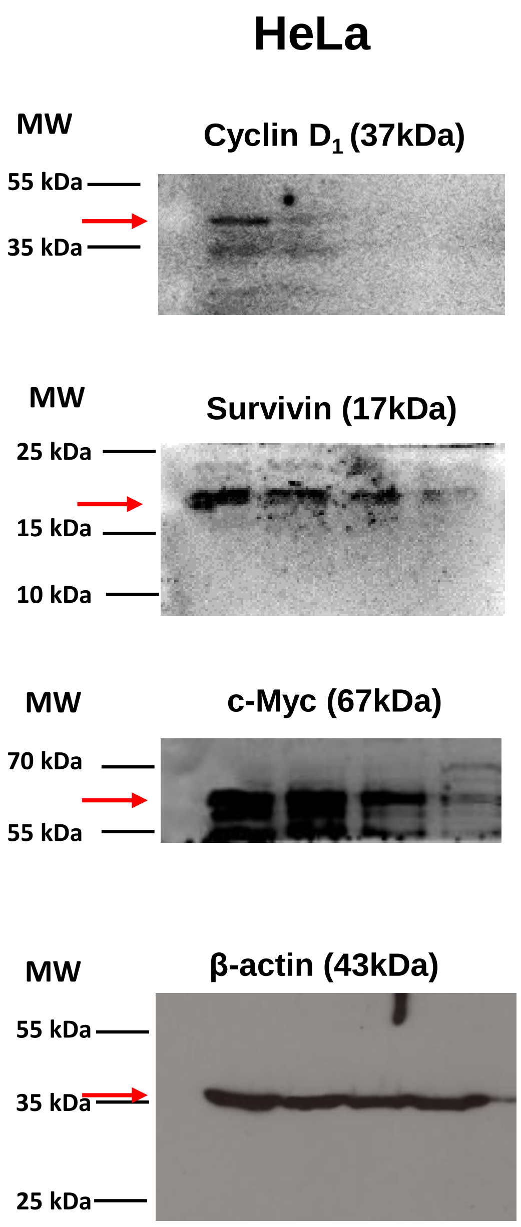

Fig.3.b-HeLa

Gel name : TMS-TMF-4f_hela cyclin d1 (Raw 1-D Image)							
Index	Name	Volume	Adj. Vol.	% Adj. Vo	Concentration		
		INT*mm2	INT*mm2				
	(uM)					cyclinD1/actin	Ratio
1	0	3434.689	3434.689	51.04619	N/A	1.05357874	1
2	2.5	1808.369	1808.369	26.87591	N/A	0.562163171	0.533575
3	5	783.9125	783.9125	11.65047	N/A	0.255309946	0.242326
4	10	701.6187	701.6187	10.42743	N/A	0.202126957	0.191848

Gel name : TMS-TMF-4f_hela survivin (Raw 1-D Image)							
Index	Name	Volume	Adj. Vol.	% Adj. Vo	Concentration		
		INT*mm2	INT*mm2				
	(uM)					survivin/actin	Ratio
1	0	4120.336	4120.336	31.55236	N/A	1.2741059	1
2	2.5	3731.29	3731.29	28.57315	N/A	1.132838929	0.889125
3	5	3347.75	3347.75	25.63611	N/A	1.038411281	0.815012
4	10	1859.351	1859.351	14.23838	N/A	0.52098631	0.408903

Gel name : TMS-TMF-4f_hela c-myc (Raw 1-D Image)							
Index	Name	Volume	Adj. Vol.	% Adj. Vo	Concentration		
		INT*mm2	INT*mm2				
	(uM)					c-Myc/actin	Ratio
1	0	6451.973	6451.973	27.127	N/A	1.99394574	1
2	2.5	6530.109	6530.109	27.45551	N/A	2.013035718	1.009574
3	5	6196.518	6196.518	26.05295	N/A	1.973521352	0.989757
4	10	4605.726	4605.726	19.36454	N/A	1.335423439	0.669739

Gel name : TMS-TMF-4f_hela bactin (Raw 1-D Image)					
Index	Name	Volume	Adj. Vol.	% Adj. Vo	Concentration
		INT*mm2	INT*mm2		
	(uM)				
1	0	3614.301	4152.766	27.59569	N/A
2	2.5	3614.301	3614.301	24.01752	N/A
3	5	3550.244	3550.244	23.59185	N/A
4	10	3731.29	3731.29	24.79493	N/A

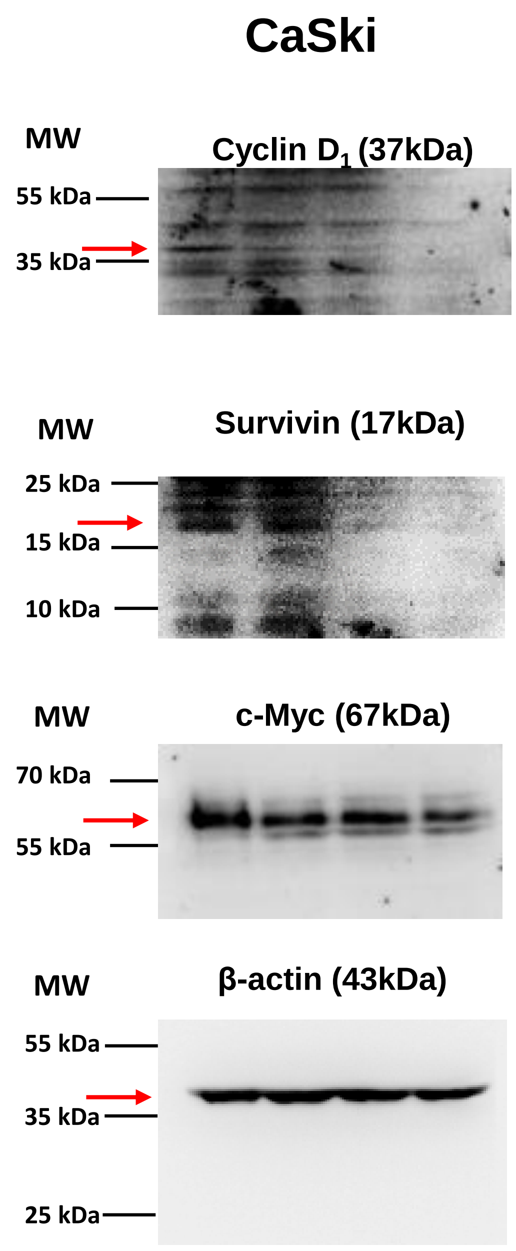

Fig.3.b-CaSki

Gel name : TMS-TMF-4f_caski cyclin d1 (Raw 1-D Image)							
Index	Name	Volume	Adj. Vol.	% Adj. Vo	Concentration		
		INT*mm2	INT*mm2				
	(uM)					cyclinD1/actin	Ratio
1	0	2293.214	2293.214	39.94985	N/A	0.551602163	1
2	2.5	1878.19	1878.19	32.71975	N/A	0.402803441	0.730243
3	5	1060.07	1060.07	18.46736	N/A	0.233508117	0.423327
4	10	508.7589	508.7589	8.863036	N/A	0.126922109	0.230097

Gel name : TMS-TMF-4f_caski survivin (Raw 1-D Image)							
Index	Name	Volume	Adj. Vol.	% Adj. Vo	Concentration		
		INT*mm2	INT*mm2				
	(uM)					survivin/actin	Ratio
1	0	3238.646	3238.646	37.98768	N/A	0.822183482	1
2	2.5	3839.964	3839.964	45.04083	N/A	0.890138524	1.082652
3	5	1035.267	1035.267	12.14316	N/A	0.239015498	0.290708
4	10	411.6408	411.6408	4.828338	N/A	0.112626401	0.136985

Gel name : TMS-TMF-4f_caski c-myc (Raw 1-D Image)							
Index	Name	Volume	Adj. Vol.	% Adj. Vo	Concentration		
		INT*mm2	INT*mm2				
	(uM)					c-Myc/actin	Ratio
1	0	4559.876	4559.876	30.9181	N/A	1.226497134	1
2	2.5	3638.76	3638.76	24.6725	N/A	0.90926595	0.741352
3	5	3948.15	3948.15	26.77031	N/A	1.013043415	0.825965
4	10	2601.457	2601.457	17.6391	N/A	0.770287048	0.628038

Gel name : TMS-TMF-4f_caski bactin (Raw 1-D Image)					
Index	Name	Volume	Adj. Vol.	% Adj. Vo	Concentration
		INT*mm2	INT*mm2		
	(uM)				
1	0	3689.771	3689.771	24.84333	N/A
2	2.5	3953.856	3953.856	26.62143	N/A
3	5	3840.881	3840.881	25.86076	N/A
4	10	3367.649	3367.649	22.67448	N/A

Figure 3c

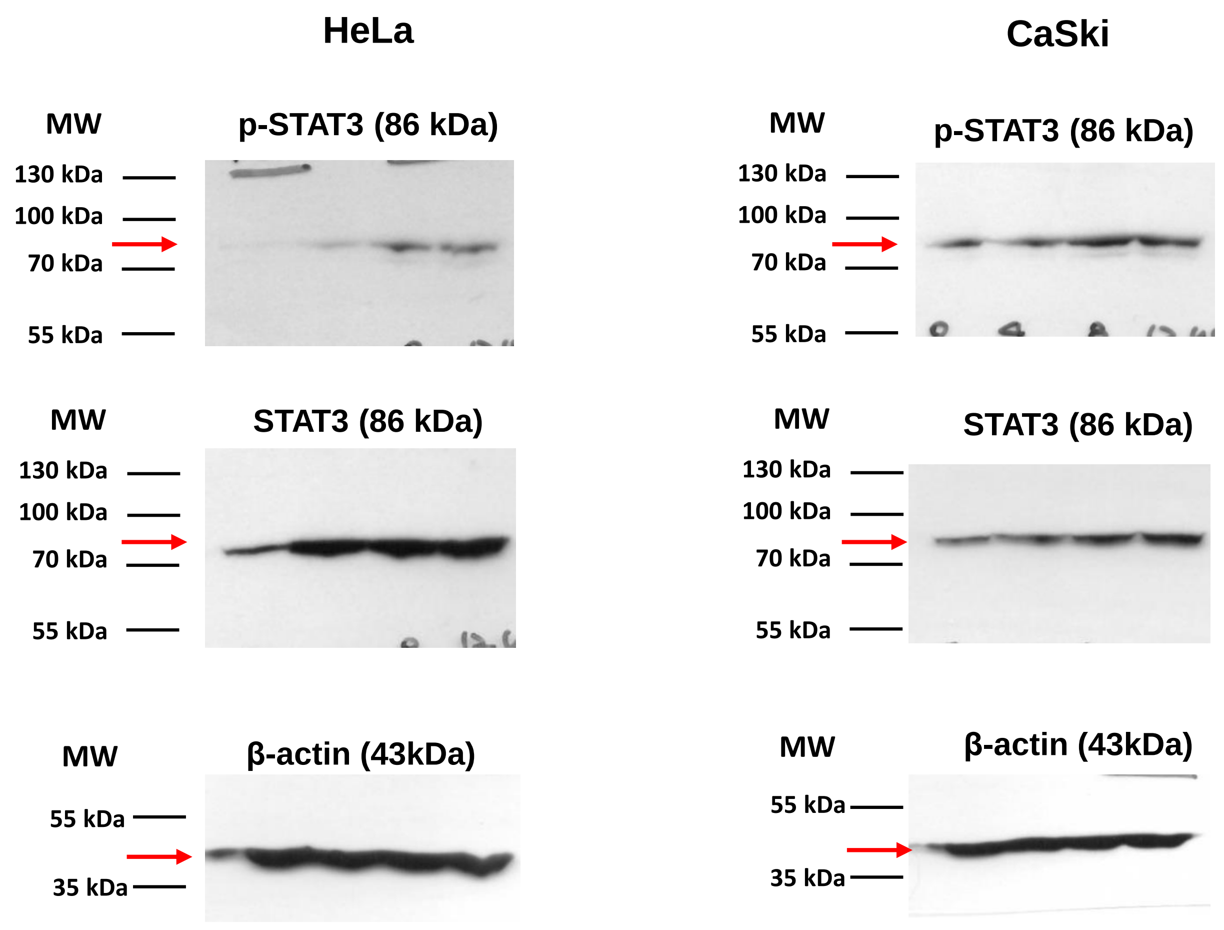

Figure 3d

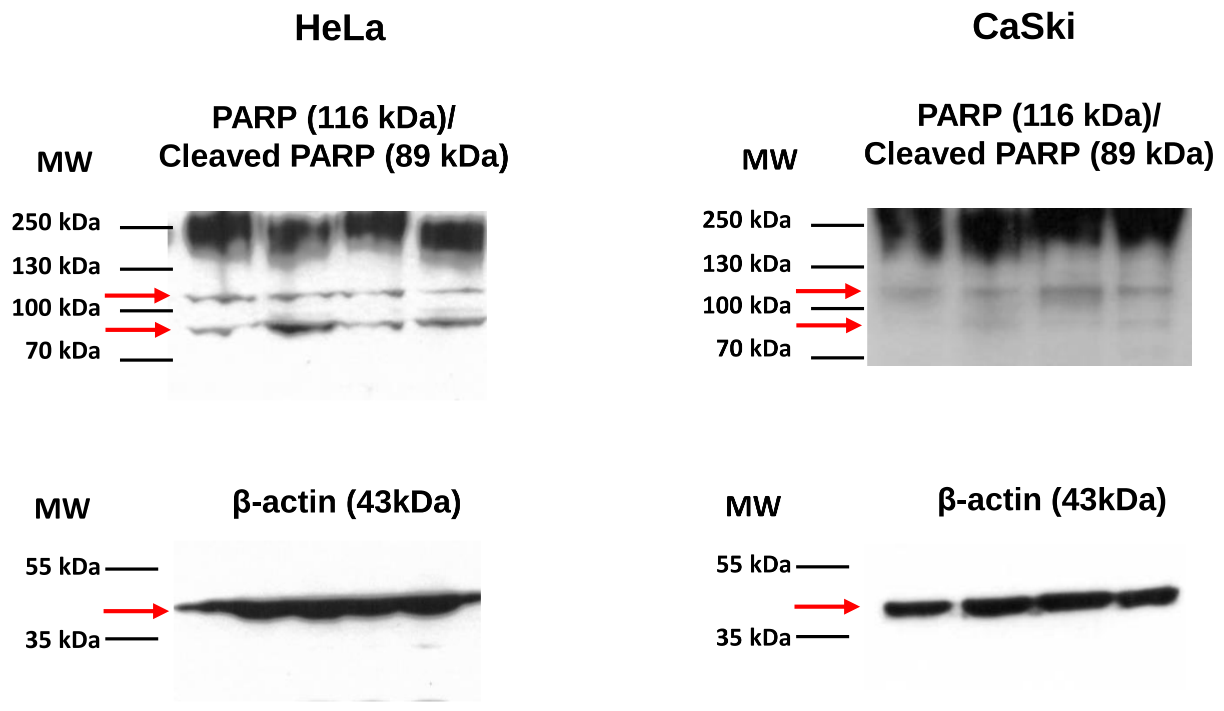

Figure 4b

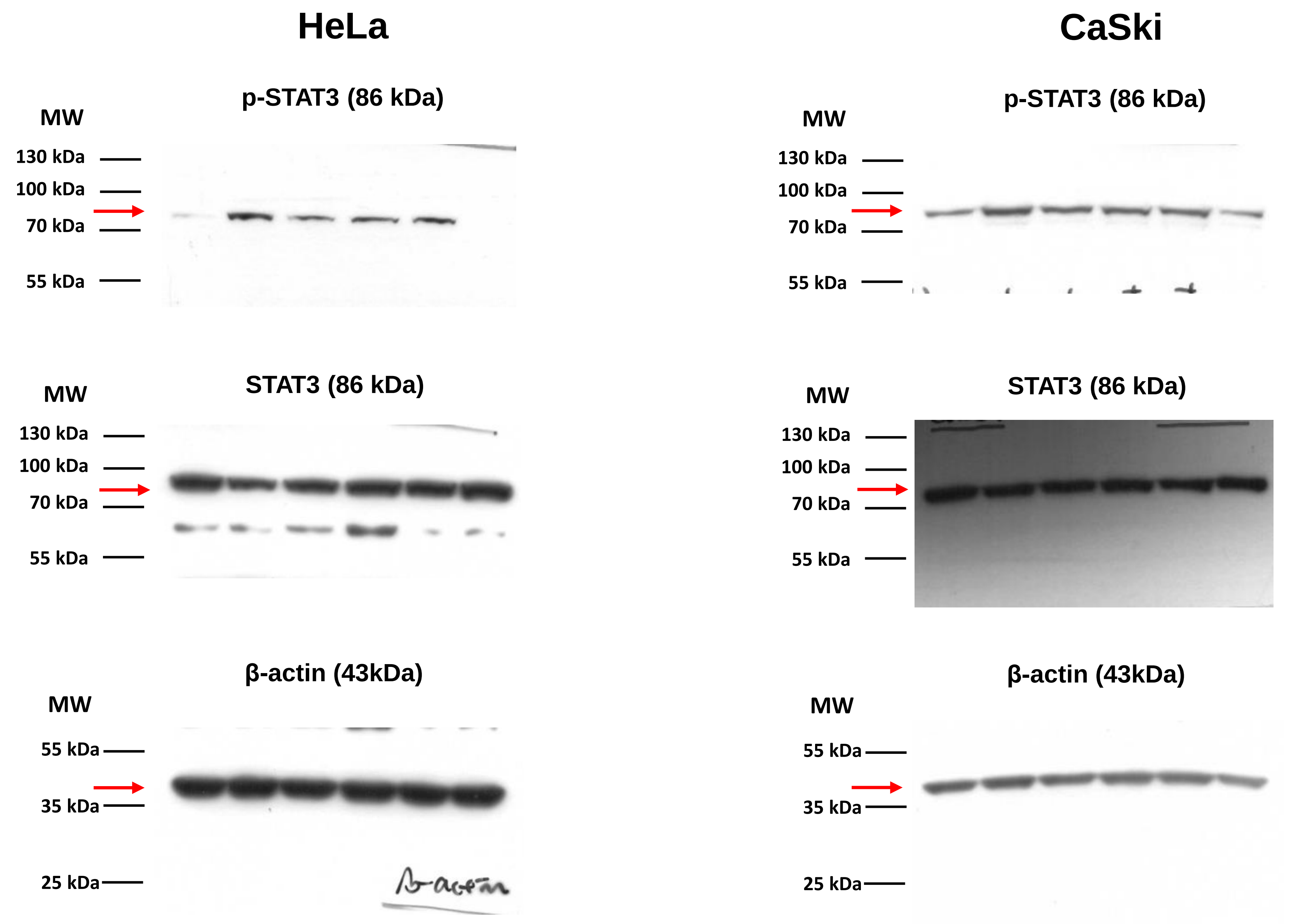

Gel name : IL-6-p-STAT3-HeLa (Raw 1-D Image)							
Index	Name	Volume	Adj. Vol.	% Adj. Vo	Concentration		
		INT*mm2	INT*mm2				
IL-6	TMS-TMF-4f					p-STAT3/STAT3	Ratio
0	0	0	0	0	N/A	0	0
10	0	2073.498	2073.498	35.10905	N/A	2.832960011	1
10	5	1060.581	1060.581	17.95807	N/A	1.248993	0.44
10	5	1311.601	1311.601	22.20841	N/A	1.2051586	0.43
10	10	1317.824	1317.824	22.31377	N/A	1.17805566	0.42
10	10	142.3733	142.3733	2.410705	N/A	0.124265744	0.04

Gel name : IL-6-STAT3-HeLa (Raw 1-D Image)							
Index	Name	Volume	Adj. Vol.	% Adj. Vo	Concentration		
		INT*mm2	INT*mm2				
IL-6	TMS-TMF-4f						
0	0	4209.595	4209.595	16.46033	N/A		
10	0	3169.423	3169.423	12.39306	N/A		
10	5	3677.064	3677.064	14.37804	N/A		
10	5	4712.755	4712.755	18.42779	N/A		
10	10	4844.052	4844.052	18.94119	N/A		
10	10	4961.286	4961.286	19.39959	N/A		

Gel name : IL-6-p-STAT3-Caski (Raw 1-D Image)							
Index	Name	Volume	Adj. Vol.	% Adj. Vo	Concentration		
		INT*mm2	INT*mm2				
IL-6	TMS-TMF-4f					p-STAT3/STAT3	Ratio
0	0	645.5334	645.5334	11.69218	N/A	0.653933107	0.38
10	0	1706.862	1706.862	30.9154	N/A	1.719610748	1
10	5	965.0021	965.0021	17.47853	N/A	0.979538897	0.57
10	5	862.578	862.578	15.62338	N/A	0.91290642	0.53
10	10	868.3028	868.3028	13.62707	N/A	0.90155955	0.52
10	10	472.7938	472.7938	8.563443	N/A	0.608649907	0.35

Gel name : IL-6-STAT3-Caski (Raw 1-D Image)							
Index	Name	Volume	Adj. Vol.	% Adj. Vo	Concentration		
		INT*mm2	INT*mm2				
IL-6	TMS-TMF-4f						
0	0	4863.591	4863.591	17.87977	N/A		
10	0	4890.348	4890.348	17.97814	N/A		
10	5	4853.759	4853.759	17.84363	N/A		
10	5	4655.258	4655.258	17.11389	N/A		
10	10	4111.526	4111.526	15.115	N/A		
10	10	3827.153	3827.153	14.06957	N/A		

Figure 7b

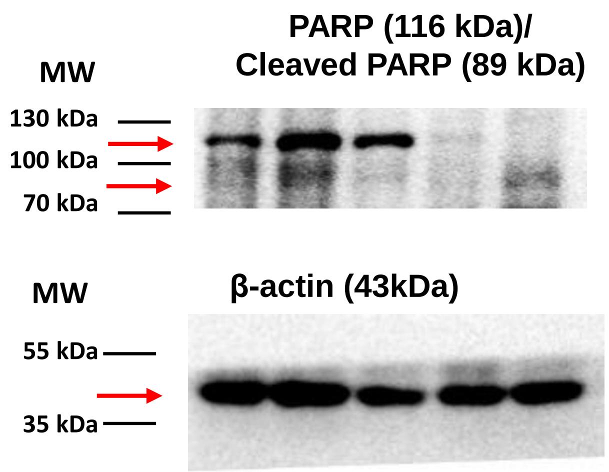

Gel name : Xeno-PARP (Raw 1-D Image)							
Index	Name	Volume	Adj. Vol.	% Adj. Vo	Concentration		
		INT*mm2	INT*mm2				
						cleaved PARP/actin	Ratio
1	con	7088.921	7088.921	15.98974	N/A	0.74	0.5
2	PTX	9994.133	9994.133	32.4115	N/A	1.39	1.0
3	TMS-TMF-4f-5mpk	3790.316	3790.316	12.29219	N/A	0.77	0.6
4	TMS-TMF-4f-10mpk	3334.198	3334.198	15.81298	N/A	0.81	0.6
5	TMS-TMF-4f-20mpk	6627.577	6627.577	21.49358	N/A	1.09	0.8

Gel name : Xeno- b-actin (Raw 1-D Image)					
Index	Name	Volume	Adj. Vol.	% Adj. Vo	Concentration
		INT*mm2	INT*mm2		
1	con	12050.08	12050.08	21.62821	N/A
2	PTX	14276.41	14276.41	23.25464	N/A
3	TMS-TMF-4f-5mpk	11012.03	11012.03	15.93734	N/A
4	TMS-TMF-4f-10mpk	11931.48	11931.48	19.43502	N/A
5	TMS-TMF-4f-20mpk	12121.64	12121.64	19.74478	N/A

Figure 7c

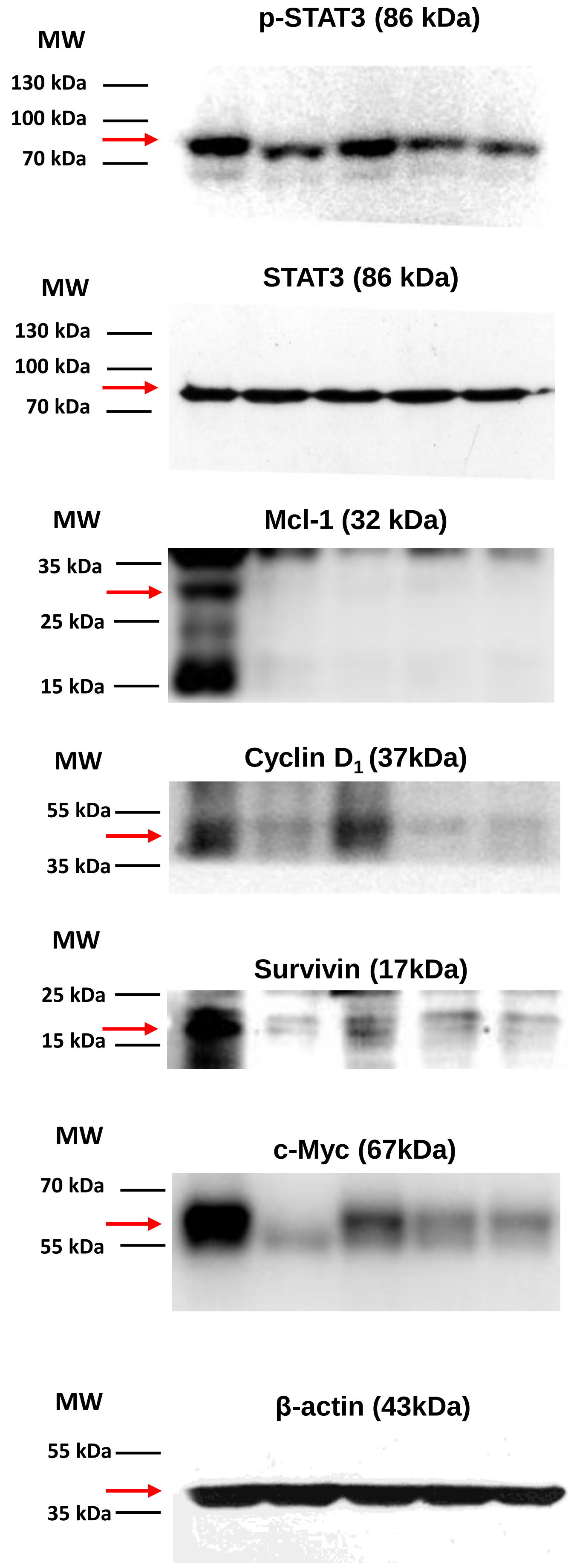

Gel name : Xeno- p-STAT3 (Raw 1-D Image)							
Index	Name	Volume	Adj. Vol.	% Adj. Vo	Concentration		
		INT*mm2	INT*mm2				
						p-STAT3/STAT3	Ratio
1	con	10972.33	10972.33	25.18791	N/A	1.30	1.0
2	PTX	7955.232	7955.232	18.26192	N/A	0.88	0.7
3	TMS-TMF-4f-5mpk	10554.29	10554.29	24.22828	N/A	1.22	0.9
4	TMS-TMF-4f-10mpk	7745.53	7745.53	17.78053	N/A	0.85	0.7
5	TMS-TMF-4f-20mpk	6334.491	6334.491	14.54137	N/A	0.75	0.6

Gel name : Xeno-STAT3 (Raw 1-D Image)					
Index	Name	Volume	Adj. Vol.	% Adj. Vo	Concentration
		INT*mm2	INT*mm2		
1	con	5913.843	5913.843	19.39369	N/A
2	PTX	6303.129	6303.129	20.6703	N/A
3	TMS-TMF-4f-5mpk	6031.823	6031.823	19.78059	N/A
4	TMS-TMF-4f-10mpk	6345.692	6345.692	20.80988	N/A
5	TMS-TMF-4f-20mpk	5899.157	5899.157	19.34553	N/A

Gel name : Xeno- Mcl-1 (Raw 1-D Image)							
Index	Name	Volume	Adj. Vol.	% Adj. Vo	Concentration		
		INT*mm2	INT*mm2				
						Mcl-1/actin	Ratio
1	con	3411.732	3411.732	6.451036	15.50139	0.76	1.0
2	PTX	11526.01	11526.01	21.79384	4.588453	0.23	0.3
3	TMS-TMF-4f-5mpk	12619.08	12619.08	23.86064	4.191002	0.21	0.3
4	TMS-TMF-4f-10mpk	12518.15	12518.15	24.6698	4.053539	0.20	0.3
5	TMS-TMF-4f-20mpk	12811.6	12811.6	26.22468	3.813202	0.20	0.3

Gel name : Xeno-cyclin D1 (Raw 1-D Image)							
Index	Name	Volume	Adj. Vol.	% Adj. Vo	Concentration		
		INT*mm2	INT*mm2				
						Cyclin D1/actin	Ratio
1	con	7940.547	7940.547	13.15289	7.602893	0.37	1.00
2	PTX	13136.55	13136.55	21.75966	4.595661	0.23	0.60
3	TMS-TMF-4f-5mpk	7579.013	7579.013	12.55404	7.965566	0.39	1.05
4	TMS-TMF-4f-10mpk	15921.79	15921.79	26.37318	3.79173	0.19	0.51
5	TMS-TMF-4f-20mpk	15793.23	15793.23	28.16024	3.551107	0.19	0.50

Gel name : Xeno- survivin (Raw 1-D Image)							
Index	Name	Volume	Adj. Vol.	% Adj. Vo	Concentration		
		INT*mm2	INT*mm2				
						survivin/actin	Ratio
1	con	3725.227	3725.227	6.659044	15.01717	0.74	1.0
2	PTX	15022.37	15022.37	24.8533	4.023611	0.20	0.3
3	TMS-TMF-4f-5mpk	10716.08	10716.08	18.15557	5.507952	0.27	0.4
4	TMS-TMF-4f-10mpk	12526.61	12526.61	22.39199	4.465883	0.22	0.3
5	TMS-TMF-4f-20mpk	13952.09	13952.09	24.9401	4.009607	0.21	0.3

Gel name : Xeno- c-Myc (Raw 1-D Image)							
Index	Name	Volume	Adj. Vol.	% Adj. Vo	Concentration		
		INT*mm2	INT*mm2				
						c-Myc/actin	Ratio
1	con	24876.12	24876.12	34.03567	N/A	1.67	1.0
2	PTX	9439.074	9439.074	12.9146	N/A	0.63	0.4
3	TMS-TMF-4f-5mpk	16415.61	16415.61	22.45995	N/A	1.10	0.7
4	TMS-TMF-4f-10mpk	11962.22	11962.22	16.36678	N/A	0.82	0.5
5	TMS-TMF-4f-20mpk	10395.36	10395.36	14.223	N/A	0.75	0.5

Gel name : Xeno-b-actin (Raw 1-D Image)					
Index	Name	Volume	Adj. Vol.	% Adj. Vo	Concentration
		INT*mm2	INT*mm2		
1	con	7347.158	7347.158	20.34314	N/A
2	PTX	7353.132	7353.132	20.35968	N/A
3	TMS-TMF-4f-5mpk	7361.097	7361.097	20.38174	N/A
4	TMS-TMF-4f-10mpk	7245.108	7245.108	20.06058	N/A
5	TMS-TMF-4f-20mpk	6809.649	6809.649	18.85486	N/A
